# Luminal hormone-responsive cells tune the regenerative remodeling of mammary glands in large mammals

**DOI:** 10.1038/s41421-025-00848-3

**Published:** 2025-12-30

**Authors:** Yongtao Li, Liping Zhang, Tao Luo, Wenying Zhang, Teng Wang, Fanming Liu, Shengda Lin, Jun Luo, Jianxin Liu, Jinrong Peng, Chaochen Wang, Wei Wang, Hengbo Shi

**Affiliations:** 1 Institute of Dairy Science, College of Animal Sciences, Zhejiang University, Hangzhou, Zhejiang China; 2 National Institute of Biological Sciences, Beijing, China; 3 Centre of Biomedical Systems and Informatics, Zhejiang University-University of Edinburgh Institute (ZJU-UoE Institute), Zhejiang University School of Medicine, International Campus, Zhejiang University, Zhejiang, China; 4 Life Sciences Institute, Zhejiang University, Hangzhou, Zhejiang China; 5 College of Animal Science and Technology, Northwest A&F University, Yangling, Shaanxi China; 6 Zhejiang Key Laboratory of Cow Genetic Improvement & Milk Quality Research, Ministry of Education Key Laboratory of Molecular Animal Nutrition, Zhejiang University, Hangzhou, Zhejiang China

**Keywords:** Self-renewal, Cell growth

## Abstract

The remodeling of mammary glands during pregnancy is essential for initiating lactation. In dairy animals, the overlap of pregnancy and mammary involution triggers a unique process, regenerative remodeling, which is critical for extending lactation duration and enhancing milk production. Unlike the complete regression of lobuloalveolar structures during involution, the regenerative remodeling preserves alveolar structures and promotes rapid mammary gland renewal. However, the cellular and molecular mechanisms underlying such process remain elusive. Here, taking dairy goats (*Capra hircus*) as a ruminant model, we identified four luminal cell populations through single-cell RNA-sequencing and found a significant reduction in luminal hormone-responsive (LumHR) cells and an increase in luminal secretory precursors (LumSecP) during regenerative remodeling. A reduction of LumHR cells during regenerative remodeling is essential for promoting the accumulation of LumSecP. Goat mammary organoids and in vivo genetic ablation assays suggested that LumHR cells function as a crucial switch for the differentiation of LumSecP to LumSec cells through the prolactin receptor pathway. Furthermore, high levels of IRF1 inhibited while downregulation of IRF1 stimulated the proliferation of LumHR cells. We showed that IRF1 regulated the dynamics of LumHR cells through hormonal signaling targets, including ESRRB. Our findings identified a key cell type responsible for the dynamics of luminal lineages during regenerative remodeling in large mammals and highlighted the potential for accelerating tissue regeneration through targeted modulation of lineage stage-specific regulators.

## Introduction

Tissue remodeling is a fundamental biological process involved in many aspects of development and regeneration. Dysregulation of remodeling usually causes developmental defects, impairment of physiological functions, and increases susceptibility to diseases^1–3^. As a critical feature for mammals, the milk-producing mammary glands undergo cyclical expansion and regression. The involvement of complex tissue remodeling is indispensable for efficient production of milk, particularly in dairy animals (e.g., cows and goats)^4,5^.

In spite of the marked differences in lifestyle and evolutionary distance, the mammary glands of dairy animals, humans, and mice are similar in physiology and developmental processes^6–8^. In adult mammals, tissue remodeling occurs at distinct reproductive stages including pregnancy, lactation, and involution^9–12^. During pregnancy, the mammary epithelium undergoes proliferation and differentiation to form lobuloalveolar structures^11,13^. Stimulated by estrous hormones such as prolactin (PRL), the lobuloalveolar cells initiate and maintain milk secretion during the lactation stage^12^. Weaning triggers a typical involution of the mammary gland, leading to a cell death-mediated loss of lobuloalveolar structures, followed by the return of a pre-pregnant state^11,13,14^. Although much research has been undertaken to understand the dynamics in luminal cells in cultured cells and rodents^10,15–17^, key questions remain unanswered about the regulation of the regular transition during cyclical mammary gland remodeling in female mammals, particularly from pregnancy to lactation in large animals.

**Fig. 1:**
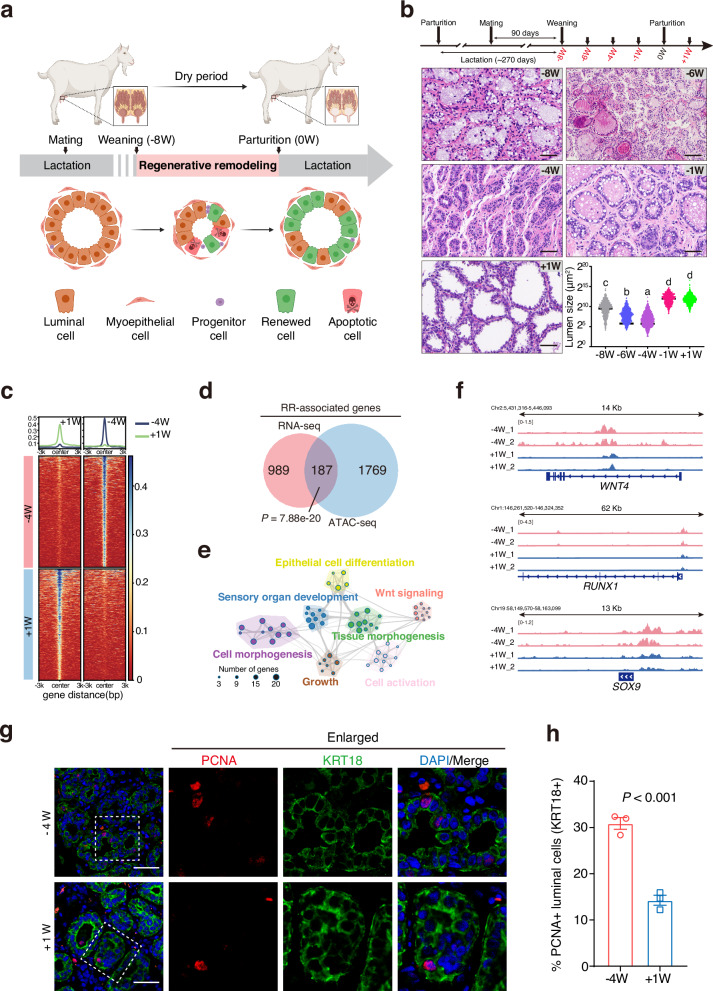
Characterization of regenerative remodeling in goat mammary glands. **a** A diagram showing the tissue renewal and dynamics in alveolar structures of goat mammary gland during regenerative remodeling (RR) (created with BioRender.com). Concurrent pregnancy and mammary involution during RR can markedly reduce the extent of mammary cell death and lead to a unique process of gland renewal essential for subsequent lactation in dairy animals. The RR generally starts after weaning, which typically occurs approximately 8 weeks before parturition (−8W) in dairy ruminants (e.g., goats). This process may involve the activity of progenitors derived from a preexisting progenitor pool. **b** H&E staining and measurement of lumen sizes in goat mammary tissues at different time points (−8W, −6W, −4W, −1W, and +1W). Lumen sizes were measured using Image J software and presented using a violin plot (*n* = 982 lumens at −8W, *n* = 1066 lumens at −6W, *n* = 1725 lumens at −4W, *n* = 582 lumens at −1W, and *n* = 1293 lumens at +1 W). An ANOVA analysis was performed for the lumen sizes. Statistical significance in ANOVA analysis (*P* < 0.05) was indicated by different letters. Scale bars, 50 μm. **c** Heatmaps showing the ATAC-seq signal intensity of top 1000 genomic loci with increased and decreased chromatin accessibility in goat mammary epithelial cells at −4W compared to +1 W. The average signal intensity is shown on top. **d** Venn diagram showing 187 RR-associated genes that were both transcriptionally upregulated and associated with regions of significantly increased chromatin accessibility in luminal cells. Only upregulated DEGs and peaks are considered. *P* = 7.88e−20 (hypergeometric test). **e** Network diagrams illustrate pathways significantly enrich for 187 RR-associated genes. Each node represents a significantly enriched term, with node size proportional to the number of contributing genes. Similar terms with a high degree of redundancy were clustered, as depicted. **f** ATAC-seq signal on three representative genes related mammary cell proliferation. **g** Representative images of immunofluorescence staining for PCNA (red), KRT18 (green), and DAPI (blue) in goat mammary tissues. Scale bar, 50 μm. **h** Bar plots exhibiting the percentage of PCNA-positive cells in luminal cells (labeled by KRT18) at −4W and +1W. *n* = 3 goats per group. The data are presented as the mean ± SEM. The *P* values of two-sided Student’s *t*-tests are shown.

The overlap of pregnancy and mammary involution occurs in various mammals including humans and rodents. Such overlap is particularly important in dairy animals (e.g., dairy cows and goats) to extend lactation duration and improve milk production efficiency. Unlike the complete regression resulting from markedly cell death during involution, the pregnancy limits this regression, thereby preserving alveolar structures and promoting rapid mammary gland renewal (Fig. 1a)^4,18,19^. We define this unique process as regenerative remodeling (RR), also referred to regenerative involution^4,5^, to distinguish it from the typical involution that occurs in mammary glands of non-pregnant mammals following the cessation of lactation. In mice, pregnancy-induced RR decreases the apoptosis rate but increases the proliferation rate of luminal cells, compared to typical involution^18^. Shortening the length of RR in dairy cows dramatically reduced the milk production in the subsequent lactation stage, highlighting that RR is critical for replacing senescent cells and the renewal of mammary glands^19–21^.

The fully developed lobuloalveolar unit of the mammary gland comprises outer basal cells and inner luminal cells that include luminal hormone-responsive (LumHR) cells and luminal secretory (LumSec) cells. Multiple transitional subtypes are present in the luminal lineages depending on their differentiation states. Recent single-cell RNA sequencing (scRNA-seq) data in rodents suggest that the subtypes and proportions of luminal cells undergo dramatic changes across distinct reproductive stages^22–27^. Although remarkably morphological changes during RR were observed, the underlying molecular mechanisms responsible for the regulation of luminal lineages in this regenerative state remain elusive, particularly in large animals.

While RR occurs in humans^28^, rodents^29,30^, and other animals^31,32^, a systematic characterization of mammary remodeling during this process is lacking. Compared with other animals, dairy goats (*Capra hircus*) are an ideal model for investigating RR in large mammals due to their availability, convenient size, and easy handling^33,34^. In this study, we performed a comprehensive single-cell analysis of RR in Saanen dairy goats and utilized goat mammary organoids to dissect the cellular and genetic programs involved in this process. We discovered an essential role of LumHR population in controlling the differentiation of luminal secretory progenitors (LumSecP) to LumSec cells through PRL receptor signaling. A reduction of LumHR cells triggered by the upregulation of IRF1-ESRRB pathway promoted the accumulation of LumSecP. Our findings highlight that targeted modulation of lineage stage-specific regulators contributes to the rapid tissue renewal in the mammary glands of large mammals.

## Results

### Morphological characterization of RR in mammary glands

The RR generally starts when lactation ceases, which typically occurs approximately 8 weeks before parturition (−8W) in dairy cows and goats (Fig. 1a). In the current study, healthy Saanen dairy goats undergoing the second lactation period were selected. To determine the critical stages of RR, mammary tissues were collected at −8W (the onset of weaning), −6W, −4W, −1W, and 1W post-parturition (+1W), which covers the entire course of RR (Fig. 1b). In contrast to the complete tissue regression in typical involution, we observed a preservation and morphological restoration of alveolar structures during RR (Supplementary Fig. S1a). Hematoxylin and eosin (H&E) staining uncovered a gradual reduction in lumen sizes from −8W to −4W, followed by a marked increase from −4W to +1 W during RR (Fig. 1b). As a result, the number of luminal cells visualized by the expression of a known marker, Keratin 18 (KRT18), displayed an evident reduction from −8W to −4W and a rapid increase from −4W to +1 W (Supplementary Fig. S1b).

To identify genes involved in RR, we performed bulk RNA-seq on mammary tissues collected at −8W, −6W, −4W, and compared their transcriptomes with those at +1 W (Supplementary Tables S1 and S2). Our data uncovered the most pronounced transcriptional changes at −4W, with 1032 genes significantly upregulated and 807 downregulated (log_2_|fold change| > 1 and adjusted *P* < 0.05, Supplementary Fig. S1c, d and Dataset S1). Notably, the differentially expressed genes (DEGs) identified at −4W were significantly enriched in biological processes related to mammary gland morphogenesis, mammary gland epithelium development and branching involved in pregnancy (Supplementary Fig. S1e). These transcriptional alterations, together with the observed reduction in lumen size at −4W compared to +1 W, suggest that −4W represents a pivotal phase during RR.

To further elucidate the transcriptional regulatory landscape of luminal epithelium underpinning this transition, we sorted the luminal cells from the goat mammary glands at −4W and +1 W and conducted ATAC-seq (Fig. 1c; Supplementary Fig. S2a, b,Tables S1 and S3). The majority of accessible chromatin regions at −4W were located in intergenic and intronic regions, and were predominantly associated with genes involved in mammary gland development (Supplementary Fig. S2c, d). By integrating DEGs from RNA-seq with differential peak-associated genes identified from ATAC-seq (|log2fold change| > 1, adjusted *P* < 0.05), we obtained a set of 187 RR-associated genes that exhibited both significant transcriptional upregulation and increased chromatin accessibility (Fig. 1d; Supplementary Dataset S2). Functional enrichment analysis indicated that these RR-associated genes were mainly involved in epithelial development, tissue morphogenesis and Wnt signaling pathway (Fig. 1e; Supplementary Dataset S3). Among them, 69 genes were known regulators involved in mammary gland morphogenesis and cell proliferation, including *SOX9*^35,36^, *WNT4*^37,38^, and *RUNX1*^39,40^ (Fig. 1f; Supplementary Fig. S2e, f, and Dataset S2). These regulators were primarily upregulated at −4W, which is consistent with the significantly elevated ratio of proliferating cell nuclear antigen (PCNA)-positive luminal cells (labeled by KRT18) at −4W (Fig. 1g, h). Altogether, the morphological alterations and the identification of RR-associated transcriptional program highlight −4W as a critical transitional phase during RR.

### Identification and dynamics of luminal cells during RR

**Fig. 2:**
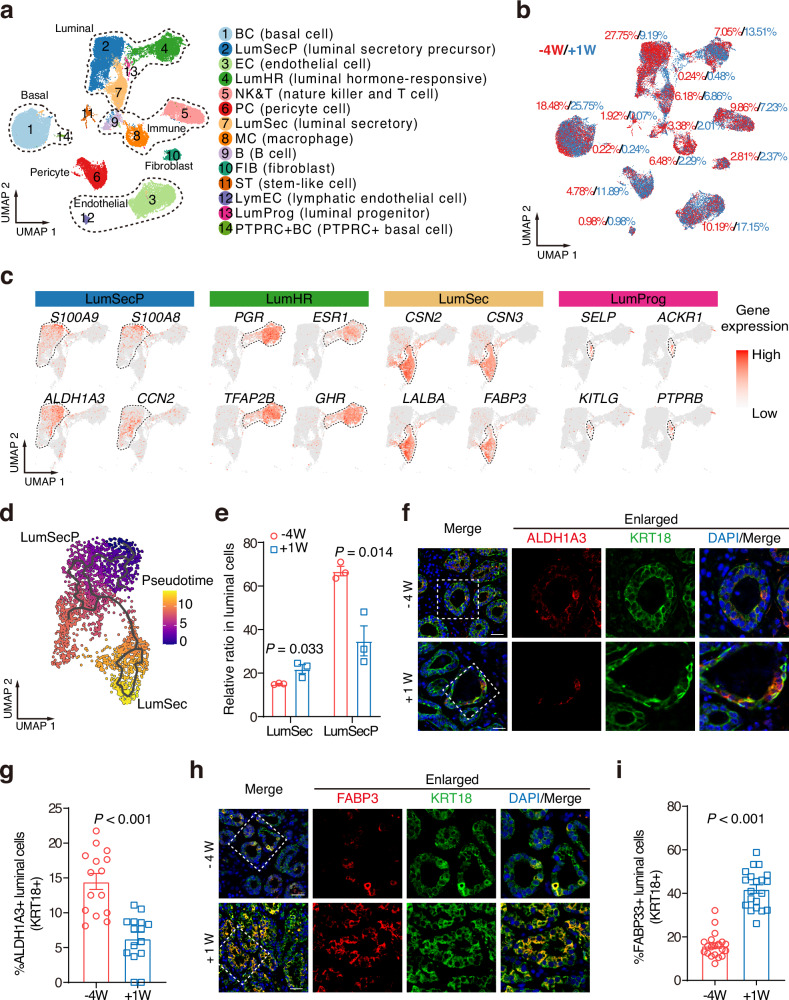
Dynamic response of luminal cell populations identified by scRNA-seq during RR. **a** UMAP plot displaying 14 identified cell types within the goat mammary glands. Cells are annotated and colored by type. Subsets of cell types including luminal, basal, fibroblast, immune, endothelial cell are labeled. **b** The percentages of −4W (red) and +1 W (blue) cells in each cell type are shown in a UMAP plot. **c** UMAP plots showing the expression of selected marker genes in four luminal subtypes. **d** Pseudotemporal trajectory analysis of scRNA-seq data of luminal secretory cells is shown in a UMAP plot. **e** Changes in the proportion of LumSecP and LumSec cells in luminal cell types were identified by scRNA-seq data at −4W and +1 W. *n* = 3 goats per group. **f** Representative images of tissue immunofluorescence staining for ALDH1A3 (red), KRT18 (green), and DAPI at −4W and +1 W. Scale bars, 50 μm. **g** Bar plots exhibiting the percentage of ALDH1A3-positive cells in luminal cells (labeled by KRT18) in **f**. *n* = 15 sections per group. **h** Representative images of tissue immunofluorescence staining for FABP3 (red), KRT18 (green), and DAPI at −4W and +1 W. Scale bars, 50 μm. **i** Bar plots exhibiting the percentage of FABP3-positive cells in luminal cells (labeled by KRT18) in **h**. *n* = 20 sections per group. The data are presented as the mean ± SEM. The *P* values of two-sided Student’s *t*-tests are shown in **e**, **g**, **i**.

To precisely dissect the progression of RR in vivo, we collected mammary tissues of Saanen dairy goats at −4W and +1 W, and performed scRNA-seq (Supplementary Fig. S3a and Tables S1 and S3). After filtering out low-quality cells, we projected 40,973 cells using the uniform manifold approximation and projection (UMAP, Supplementary Fig. S3a and Dataset S4). Using canonical markers identified in cows, mice, and humans^22,23,25,41–43^, we identified 14 distinct cell populations derived from the major cell types, including basal, luminal, endothelial, immune, pericyte cells, and fibroblasts (Fig. 2a; Supplementary Fig. S3b–e and Datasets S5 and S6). Epithelial cell types such as luminal cells (anti-KRT8) and basal cells (anti-KRT17 and anti-KRT14) were further validated using immunofluorescence staining (Supplementary Fig. S3f). Notably, integrative analysis of scRNA-seq dataset suggested that mammary tissues at −4W and +1 W shared all identified cell types but displayed profound changes in cell ratios (Fig. 2b).

The luminal cells were classified into four subpopulations: LumHR cells, LumSec cells, luminal progenitors, and LumSecP. LumSecP were identified by their enriched expression of progenitor-associated markers (e.g., *ALDH1A3*, *SA100A8, SA100A9*, *and CCN2*) and diminished expression of secretory markers for LumSec cell markers (e.g., *CSN2*, *CSN3*, *LALBA*, *and FABP3*, Fig. 2c; Supplementary Fig. S4a, b). The LumHR cells were defined by the high expression of hormone-responsive genes such as progesterone receptor (*PGR*), estrogen receptor 1 (*ESR1*), and growth hormone receptor (*GHR*, Fig. 2c; Supplementary Fig. S4a). Notably, LumHR cells showed relatively low expression of genes involved in milk protein (e.g., *CSN2*, *CSN3*, *LALBA*) and fat synthesis (e.g., *CD36*, *BTN1A1*, and *LPL*) and high levels of hormone-induced cytokines (e.g., *TNFSF11*, Supplementary Fig. S4a, b), indicating that LumHR cells primarily function as regulators of mammary development rather than direct mediators of milk secretion. Consistently, the cellular trajectory reconstruction analysis indicated that luminal progenitors expressing progenitor markers (e.g.*, KITLG, SELP, ACKR1*, and *PTPRB*) possessed the highest developmental potential (less differentiated), followed by LumHR cells and LumSecP at −4W (Fig. 2c; Supplementary Fig. S4c). GO term enrichment analysis suggested that the marker genes of each luminal cell type are highly correlated with the corresponding physiological functions of each cell type (Supplementary Fig. S4d). For example, the LumSecP were associated with positive regulation of cell population proliferation while LumHR cells were associated with differentiation and the response to steroid hormone.

The significant increase in lumen size from −4W (minimum size) to +1W (maximum size) and the change of luminal cell ratios in the scRNA-seq dataset suggest an alteration in luminal lineage compositions during this phase. To assess this idea, we conducted the lineage analysis for all luminal cells. The observation of a hybrid state for LumSecP implies a transitional stage in the differentiating process towards LumSec cells (Supplementary Fig. S5a). To validate this argument, we reconstructed the lineage dynamics between LumSecP and LumSec cells by using pseudotemporal analyses^44^. The results revealed a primary trajectory connecting LumSecP and LumSec cells with minor paths branching off (Fig. 2d). The transitional change of pseudotime-ordered genes associated with secretory lineage differentiation between these two cell subpopulations confirmed a continuous trajectory from LumSecP to LumSec cells during RR (Supplementary Fig. S5b, c). For example, the expression of precursor marker *ALDH1A3* is mainly present in the LumSecP while the luminal differential markers, including *FABP3, CSN2, CSN3, and BTN1A1* are mainly expressed in LumSec cells (Supplementary Fig. S5d). Next, we compared the dynamic changes of LumSecP in correlation with lumen size dynamics from −4W to +1 W. The percentage of LumSecP was reduced by half (*P* = 0.014) at +1 W, while the percentage of LumSec cells increased significantly (*P* = 0.033) at +1 W (Fig. 2e). In line with the dynamic changes of LumSecP and LumSec, we observed a higher percentage of ALDH1A3-positive (Fig. 2f, g, *P* < 0.001) while a lower percentage of FABP3-positive (Fig. 2h, i, *P* < 0.001) luminal cells (KRT18+) at −4W compared with that at +1 W. These findings suggest that RR is characterized by the accumulation of LumSecP. This accumulation acts as a reservoir that allows for a rapid expansion of the LumSec cells after parturition.

### LumHR cells stimulate the differentiation of LumSecP

**Fig. 3:**
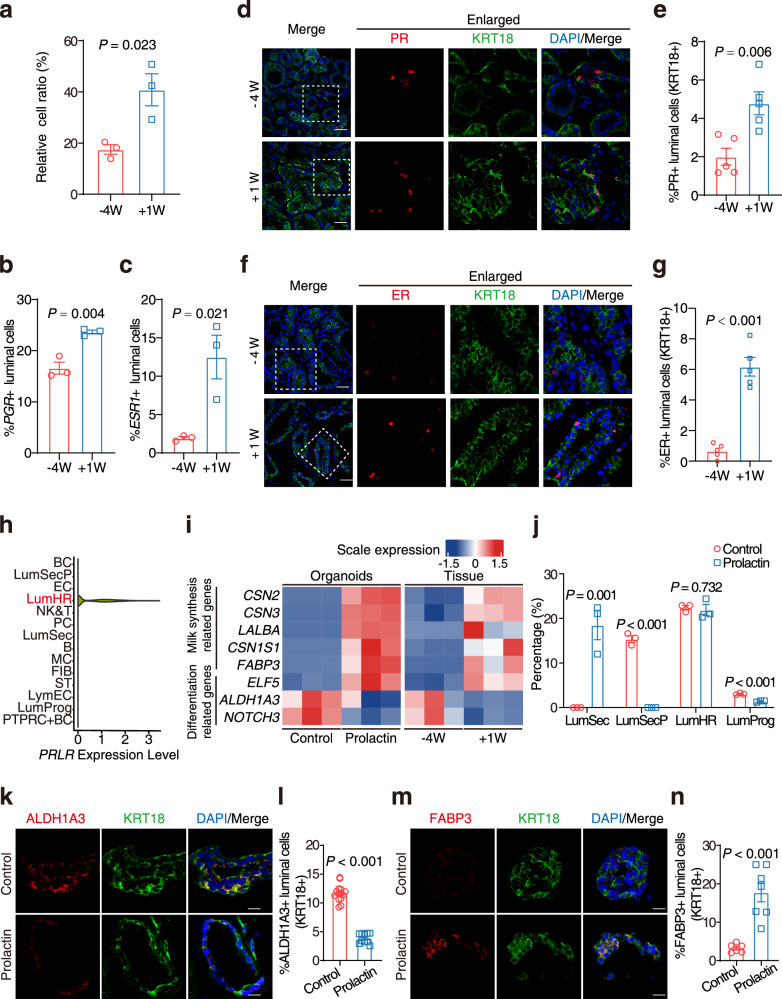
LumHR cells control the differentiation of LumSecP through the PRLR pathway. **a** Relative proportions of LumHR cells in total luminal cells identified by scRNA-seq data at −4W and +1 W. *n* = 3 goats per group. **b**, **c** Bar plots exhibiting the percentage of *PGR*- and *ESR1*-positive cells within luminal cells in scRNA-seq data. *n* = 3 goats per group. **d** Representative images of immunofluorescence staining for PR (red), KRT18 (green), and DAPI (blue). Scale bars, 50 μm. **e** Bar plots exhibiting the percentage of PR-positive cells in luminal cells (labeled by KRT18) in (**d**). *n* = 5 goats per group. **f** Representative images of immunofluorescence staining for ER (red), KRT18 (green), and DAPI (blue). Scale bars, 50 μm. **g** Bar plots exhibiting the percentage of ER-positive cells in luminal cells (labeled by KRT18) in (**f**). *n* = 5 goats. **h** Violin plot showing the specific expression of *PRLR* in LumHR cells by scRNA-seq. **i** Heatmap displaying the transcriptional level of indicated genes related to milk protein and luminal differentiation in the goat mammary organoids (*n* = 3 biological replicates) treated with or without prolactin and in the mammary tissue at −4W (non-lactation) and +1 W (lactation). *n* = 3 goats for tissues. **j** Proportions of luminal cell types in goat mammary organoids incubated with or without prolactin predicted by CIBERSORTx deconvolution. *n* = 3 biological replicates. **k** Representative images of immunofluorescence staining for ALDH1A3 (red), KRT18 (green), and DAPI in mammary organoids incubated with or without prolactin. Scale bars, 10 μm. **l** Bar plots exhibiting the percentage of ALDH1A3-positive cells in luminal cells (labeled by KRT18) in (**k**). *n* = 14 domes in the control group and *n* = 10 domes in the prolactin treated group. **m** Representative images of immunofluorescence staining for FABP3 (red), KRT18 (green) and DAPI in mammary organoids incubated with or without prolactin. Scale bars, 10 μm. **n** Bar plots exhibiting the percentage of FABP3-positive cells in luminal cells (labeled by KRT18) in (**m**). *n* = 6 domes in the control group and *n* = 7 domes in the prolactin-treated group. The data are presented as the mean ± SEM. The *P* values of two-sided Student’s *t*-tests are shown in (**a**–**c**, **e**, **g**, **j**, **l**, **n**).

The DEGs (1176 up-regulated and 942 down-regulated, referring to Supplementary Fig. S1c, d) detected in all stages of RR were defined as an RR-associated program. To determine cells deploying the identified transcriptional program involved in RR, we examined the expression of upregulated genes in this program using the single-cell dataset. We found that the activation of DEGs is mainly enriched by LumHR cells, LumSecP, and luminal progenitors (Supplementary Fig. S6a, b). Eighty-seven genes in the program were specific markers of different cell types (Supplementary Dataset S7). Twenty of them are expressed in LumHR cells and are associated with tissue development and sensory organ morphogenesis (Supplementary Fig. S6c–e). Consistent with such molecular phenotype, we found a 2-fold increase in the proportion of LumHR cells from −4W to +1W (Fig. 3a) as well as the *PGR*- and *ESR1-*positive cells (Fig. 3b, c) in luminal populations as revealed by the scRNA-seq data. Immunofluorescence staining using anti-estrogen receptor (ER) and anti-progesterone receptor (PR) antibodies further confirmed the significant increase in the signaling activities in LunHR cells from −4W to +1 W (6-fold for anti-ER and 2-fold for anti-PR, Fig. 3d–g). These data highlight a high reduction of LumHR cells during RR, which may suggest a potential role in regulating the progression of this process.

While the LumHR cells are considered a signal carrier for mammary gland development^45,46^, their role in tuning the dynamics of luminal lineage is largely unknown. Since the relative proportion of LumHR cells and LumSecP are negatively correlated, we asked whether LumHR cells regulate the differentiation of LumSecP. We investigated our scRNA-seq dataset and observed that LumHR cells are the only luminal cell population expressing *PRLR*, a known hormone receptor that induces lactation in the mammary gland (Fig. 3h). To address this question, the goat mammary organoids were established following our recent protocol (Supplementary Fig. S7a)^47^. The organoids cultured within an extracellular matrix gel maintained a bilayer structure that closely resembled the native architecture of mammary tissue (Supplementary Fig. S7b, c). The LumHR cells were activated by prolactin treatment in mammary organoids to assess the changes in luminal cells. Consistently, the lactating potential of the mammary organoids with prolactin treatment was supported by the evident accumulation of milk fat droplets stained by BODIPY (green, Supplementary Fig. S7d, e). Bulk RNA-seq analysis of mammary organoids with or without prolactin incubation showed that the genes related to milk synthesis (e.g., *FABP3*, *CNS2*, and *CNS3*) were significantly increased while the expression of progenitor markers (e.g., *ALDH1A3* and *NOTCH3*) was diminished in the prolactin-treated groups (Fig. 3i). It is noteworthy that the expression of these lactation-associated genes and 69 known cell proliferation-associated genes (Supplementary Fig. S2e) are regulated by prolactin treatment in organoids, which is consistent with the in vivo observation at −4W and +1W (Fig. 3i; Supplementary Fig. S7f, g). These findings indicate that prolactin-driven programs are present in mammary organoids, which can mimic key processes of RR in vitro.

Further, we collected goat mammary organoids treated with or without prolactin for scRNA-seq analysis (Supplementary Table S3 and Dataset S8). This analysis revealed that all four luminal cell types previously identified in mammary tissue were also represented in the organoid system (Supplementary Fig. S8a–d). Notably, PRLR was expressed in LumHR cells (Supplementary Fig. S8e–g), confirming that the organoid culture preserves the capacity for prolactin responsiveness. If LumHR cells control the differentiation of LumSecP towards LumSec, we predict that the number of LumSecP decreases while LumSec cells increase upon prolactin treatment. As predicted, the cell composition in the organoids estimated by bulk RNA-seq and deconvolution analysis showed a significant reduction in LumSecP (~15.5% to 0) and a dramatic increase in LumSec cells (0 to ~18.4%) (Fig. 3j). The shifts in cell compositions were confirmed by the scRNA-seq data from organoids (Supplementary Fig. S8d). This was further supported by the observations that the ALDH1A3-positive (Fig. 3k, l) luminal cells (KRT18+) are significantly decreased while the FABP3-positive (Fig. 3m, n) luminal cells (KRT18+) are significantly increased in the mammary organoids incubated with prolactin compared with the control group (without prolactin). Collectively, these data suggest a critical role of LumHR cells in controlling the differentiation of LumSecP to LumSec cells through PRL-PRLR pathway and imply that a low proportion of LumHR cells at –4W is essential for preventing the pre-maturation of LumSecP ahead of parturition.

### Ablation of LumHR cells reduced the differentiation of LumSecP

To establish the functional equivalence of luminal populations between goats and mice, we conducted a cross-species comparison using publicly available mouse mammary scRNA-seq datasets. This analysis revealed a relatively high degree of transcriptional conservation between goat and mouse luminal subsets (Supplementary Fig. S9a–d). In particular, goat LumHR cells shared considerable similarities in markers with the mouse LumHR cells, supporting their comparable functional identity (Supplementary Fig. S9e). Consistent with these transcriptomic findings, immunofluorescence co-staining of PR and KRT18 in mouse mammary tissue confirmed the presence of LumHR cells (Supplementary Fig. S9f).

To further investigate the functional significance of LumHR cells in the differentiation of LumSecP towards LumSec, we developed an RR model in mice and performed targeted cell ablation in vivo. To induce an RR status consistent with that in goats, lactating mice cohabited with males on day 10 of lactation, and pups were removed on lactation day 14 to induce mammary involution^18^. We found that RR-induced mice had a preservation of lobuloalveolar structures (Supplementary Fig. S10a). We engineered an adeno-associated virus (AAV) serotype 9 vector carrying the p*Prlr*-Cre expression cassette (Supplementary Fig. S10b) and a control vector expressing a non-functional stuffer sequence. *AAV-pPrlr-Cre* (1.3 × 10^11^ GC per gland) was injected into the lactating mammary glands of heterozygous *H11-CAG-LSL-ZsGreen* reporter mice on lactation day 8 to validate the expression of Cre recombinase (Supplementary Fig. S10c). The co-localization of ZsGreen- and PR-positive cells confirmed the specificity of *AAV-pPrlr-Cre* in LumHR cells (Supplementary Fig. S10d).

**Fig. 4:**
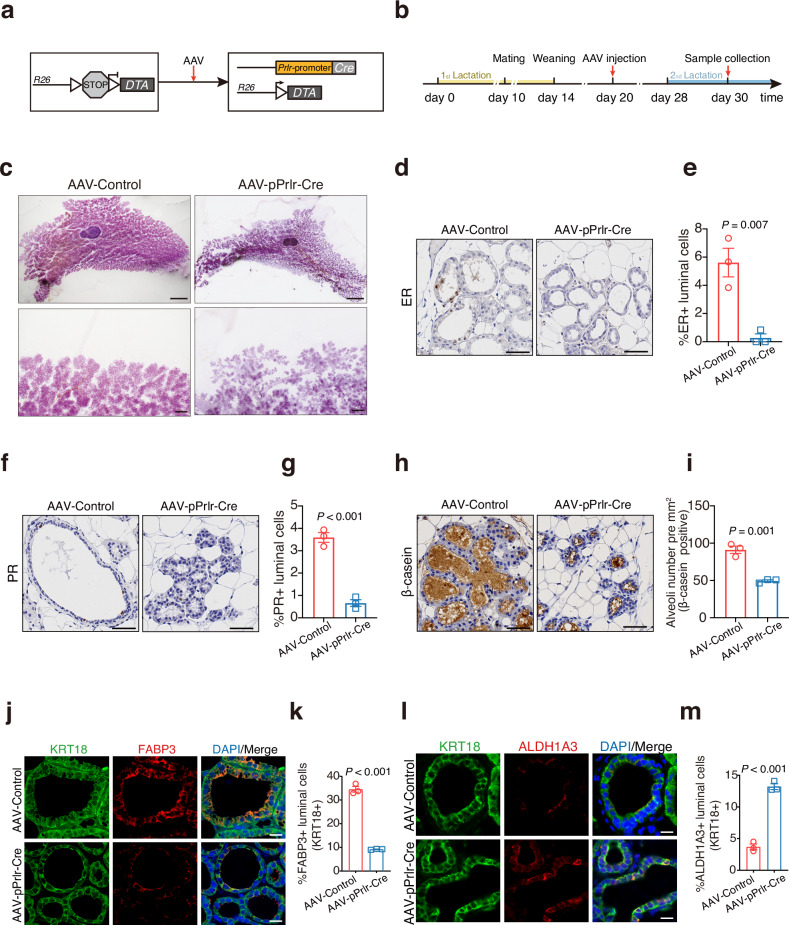
Ablation of LumHR cells disrupts the differentiation of LumSecP to LumSec cells. **a** Schematic illustration of targeted ablation of LumHR cells using the *prlr*-promoter to drive expression of DTA. **b** Experimental setup used in AAV intraductally injected mammary gland of *ROSA-DTA*^+/*−*^ mice under RR. **c** Whole-mount staining with carmine alum of mammary glands from *ROSA-DTA*^+/*−*^ mice (lactation day 2) intraductally injected with *AAV-pPrlr-Cre* or *AAV-Control*. Scale bars, 4 mm (top) and 500 μm (bottom). **d**, **e** Immunohistochemical staining and quantification of ER-positive luminal cells in mammary glands intraductally injected with *AAV-pPrlr-Cre* or *AAV-Control.* *n* = 3 mice per group. Scale bars, 50 μm. **f**, **g** Immunohistochemical staining and quantification of PR-positive luminal cells in mammary glands intraductally injected with *AAV-pPrlr-Cre* or *AAV-Control*. *n* = 3 mice per group. Scale bars, 50 μm. **h**, **i** Immunohistochemical staining and quantification of β-casein-positive alveoli number per mm^2^ in mammary glands intraductally injected with *AAV-pPrlr-Cre* or *AAV-Control*. *n* = 3 mice per group. Scale bars, 50 μm. **j** Representative images of immunofluorescence staining for FABP3 (red), KRT18 (green), and DAPI in mammary glands intraductally injected with *AAV-pPrlr-Cre* or *AAV-Control*. Scale bars, 50 μm. **k** Bar plots exhibiting the percentage of FABP3-positive cells in luminal cells (labeled by KRT18) in **j**. *n* = 3 mice per group. **l** Representative images of immunofluorescence staining for ALDH1A3 (red), KRT18 (green), and DAPI in mammary glands intraductally injected with *AAV-pPrlr-Cre* or *AAV-Contro*l. Scale bars, 50 μm. **m** Bar plots exhibiting the percentage of FABP3-positive cells in luminal cells (labeled by KRT18) in (**l**). *n* = 3 mice per group. The data are presented as the mean ± SEM. The *P* values of two-sided Student’s *t*-tests are shown in **d**, **g**, **i**, **l**, **m**.

These AAVs were intraductally injected into mammary glands of *ROSA-DTA*^+/–^ strain undergoing RR to activate the expression of diphtheria toxin (DTA) in LumHR cells (Fig. 4a,b). The depletion of LumHR cells resulted in a decrease in mammary epithelial density compared with the control mice (Fig. 4c). Immunohistochemical staining revealed a significant decrease in ER- and PR-positive luminal cells in mice injected with *AAV-pPrlr-Cre*, indicating an efficient ablation of LumHR cells (Fig. 4d–g). Consistently, the number of alveoli or alveoli expressing β-casein was significantly lower in *AAV-pPrlr-Cre* mice (Fig. 4h, i; Supplementary Fig. S10e, f). Notably, the proportion of ALDH1A3-positive luminal cells (KRT18+) was significantly increased, while that of FABP3-positive luminal cells (KRT18+) was significantly decreased following LumHR cells ablation (Fig. 4j–m). The qPCR assays further supported this shift, showing marked downregulation of luminal differentiation markers (*Foxp1*, *Areg*, *Elf5*, *Wap*, *Csn3*, and *Fabp3*) and concomitant upregulation of progenitor-associated genes (*Aldh1a3* and *S100a8*) in *AAV-pPrlr-Cre* mice (Supplementary Fig. S10g). Collectively, these findings indicate that LumHR cells are crucial for directing luminal progenitors toward secretory differentiation and highlight their role as a regulatory switch governing luminal lineage dynamics during RR.

### IRF1 negatively regulates the proliferation of LumHR cells

**Fig. 5:**
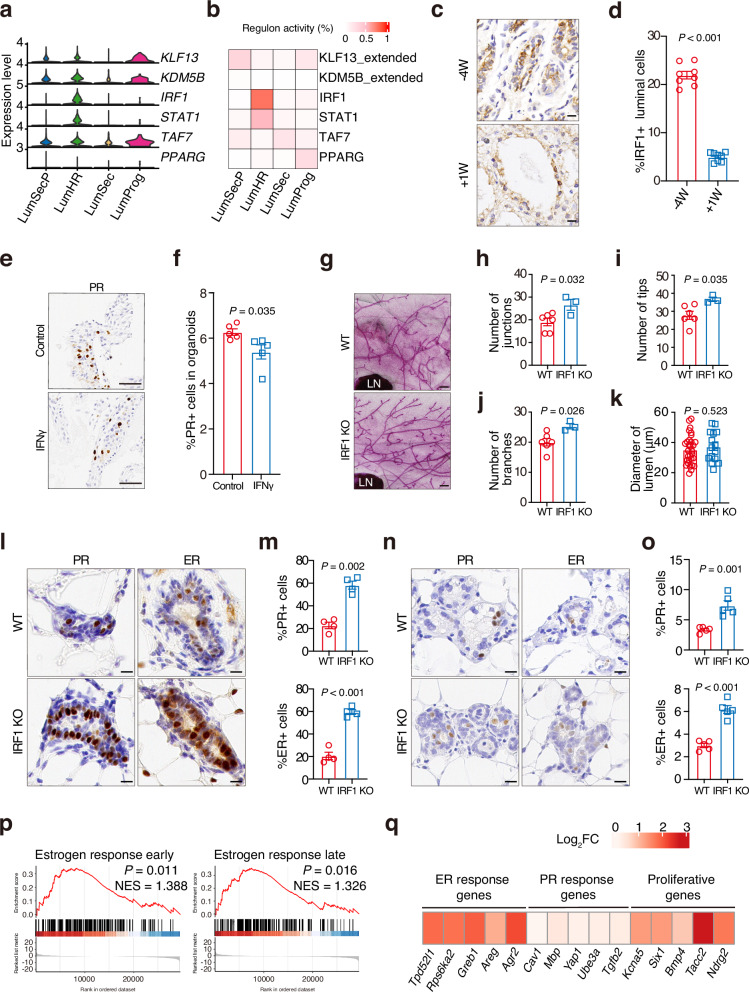
IRF1 is a novel negative regulator controlling the proliferation of LumHR cells. **a** Violin plot displaying the expression level of top transcription factors (TFs) in luminal subtypes at −4W and +1W. **b** Heatmap showing the regulon activities of the top TFs in luminal subtypes at −4W. **c** Immunohistochemical staining for IRF1 in the goat mammary gland at −4W and +1W. Nuclei were counterstained with hematoxylin. Scale bars, 50 μm. **d** Quantification of IRF1-positive cells in **c**. *n* = 8 sections from 4 goats. **e** Representative images of immunohistochemical staining for PR in the goat mammary organoids treated with or without IFNγ. Nuclei were counterstained with hematoxylin. Scale bars, 50 μm. **f** Quantification of PR-positive cells in **e**. *n* = 5 domes per group. **g** Representative images of carmine-stained mammary gland whole mounts in WT and IRF1-KO mice at 9 weeks. Scale bars, 0.4 mm. **h**−**k** Automatic quantification of the number junctions (**h**), tips (**i**), branches (**j**) and lumen diameters (**k**) of mammary tissues in **f**. *n* = 6 mice in wild type and *n* = 3 in IRF1-KO mice. *n* = 30 and *n* = 15 ductal lumens in WT and IRF1-KO mice, respectively. **l**, **m** Immunohistochemical staining (**l**) and quantification (**m**) of PR and ER in mammary tissues from WT or IRF1-KO mice at 9 weeks. Nuclei were counterstained with hematoxylin (**l**). *n* = 4 mice per group. Scale bars, 10 μm. **n**, **o** Immunohistochemical staining (**n**) and quantification (**o**) of PR and ER in mammary tissues from WT or IRF1-KO mice during RR. Nuclei were counterstained with hematoxylin. *n* = 5 mice per group. Scale bars, 10 μm. **p** Pre-ranked GSEA graphical output for the enrichment in IRF1-KO mice mammary glands of the gene set estrogen response early from the Molecular Signatures Database Hallmarks collection. *n* = 3 mice per group. **q** Heatmap representing the log_2_fold change expression of hormone-driven genes in IRF-KO compared to WT at 9 weeks. The data are presented as the mean ± SEM. The *P* values of two-sided Student’s *t*-tests are shown in **d**, **f**, **h**–**k**, **m**, **o**.

Since maintaining a low proportion of LumHR cells is essential for the accumulation of LumSecP at –4W, we asked how the expansion of LumHR cells is tightly controlled. We performed single-cell regulatory network inference and clustering analyses (SCENIC) in luminal cell populations^48^. Our analyses identified over 30 transcription factors in the luminal subsets (Supplementary Dataset S9). Among these, interferon regulatory factor 1 (*IRF1*), KLF transcription factor 13 (*KLF13*), TATA-box binding protein associated factor 7 (*TAF7*), and peroxisome proliferator activated receptor gamma (*PPARG*) were the top transcription factors within each luminal population, as determined by the regulon specificity scores (Fig. 5a; Supplementary Fig. S11a–c). Notably, IRF1 specifically displayed the highest activity in LumHR cells at the key transitional stage (−4W) of RR (Fig. 5b). Consistently, we detected significant elevations of IRF1 protein and mRNA abundance at −4W through immunohistochemical staining and qPCR (Fig. 5c, d; Supplementary Fig. S11d). Similar analyses of previously published scRNA-seq datasets also support that IRF1 is highly active in differentiated LumHR cells in mice (Supplementary Fig. S12a, b)^41^.

IRF1 is involved in apoptotic signaling in mammary epithelial cells^49–53^, which is also supported by the whole-mount staining in IRF-KO mice (Supplementary Fig. S13a). Since IFNγ is a well-established cytokine activating the IRF1 signaling^54–56^, we treated the goat mammary organoids with IFNγ (100 units/mL) to test the function of IRF1 in goats. As a result, activation of IRF1 signaling caused a significant reduction of cell proliferation in organoids (Supplementary Fig. S13b–e). In addition, the percentage of LumHR cells marked by the expression of PR decreased significantly upon IFNγ treatment (Fig. 5e, f), indicating that IRF1 signaling constrains the expansion of LumHR cells.

To investigate the in vivo function of IRF1 in LumHR cells, we examined the mammary gland of IRF1 KO mice^57^. Loss of function in IRF1 led to extended ductal invasion and increased epithelia filling to the mammary fat pad (Supplementary Fig. S13f–h). Morphologically, the mammary duct trees of IRF1-KO mice exhibited significant increases in branched ducts, junctions, and shorter tips compared with wild-type (WT) mice (Fig. 5g–k). Like RR in goats, we observed a preservation and morphological restoration of alveolar structures during RR in both WT and IRF1-KO groups (Supplementary Fig. S13i). Moreover, the mammary glands in IRF1-KO animals showed higher levels of ER- and PR-positive cells compared with WT in both virgin (Fig. 5l, m) and RR (Fig. 5n, o) stages. As expected, we observed augmented proliferation of luminal cells in IRF1-KO mice (Supplementary Fig. S14a, b), which is correlated with significantly enhanced ductal invasion.

To further characterize the molecular changes in the mutants, we carried out bulk RNA-seq analyses. The results showed that genes up-regulated in the mutants were related to tissue development and epithelium development (Supplementary Fig. S14c, d). Since hormones are essential for mammary gland development, we evaluated the gene sets associated with hormonal response. We found the “estrogen response early” and “estrogen response late” were significantly increased in IRF1-KO mammary tissue (Fig. 5p). In addition, ER target genes and their downstream proliferative genes were significantly up-regulated in the mutants (Fig. 5q; Supplementary Fig. S15a, b)^58^, indicating a negative role of IRF1 in controlling the downstream genes of steroid hormones. These findings reveal a novel role of IRF1 in constraining the expansion of LumHR cells through the integration of hormonal signaling.

### ESRRB is a target of IRF1 in LumHR cells

**Fig. 6:**
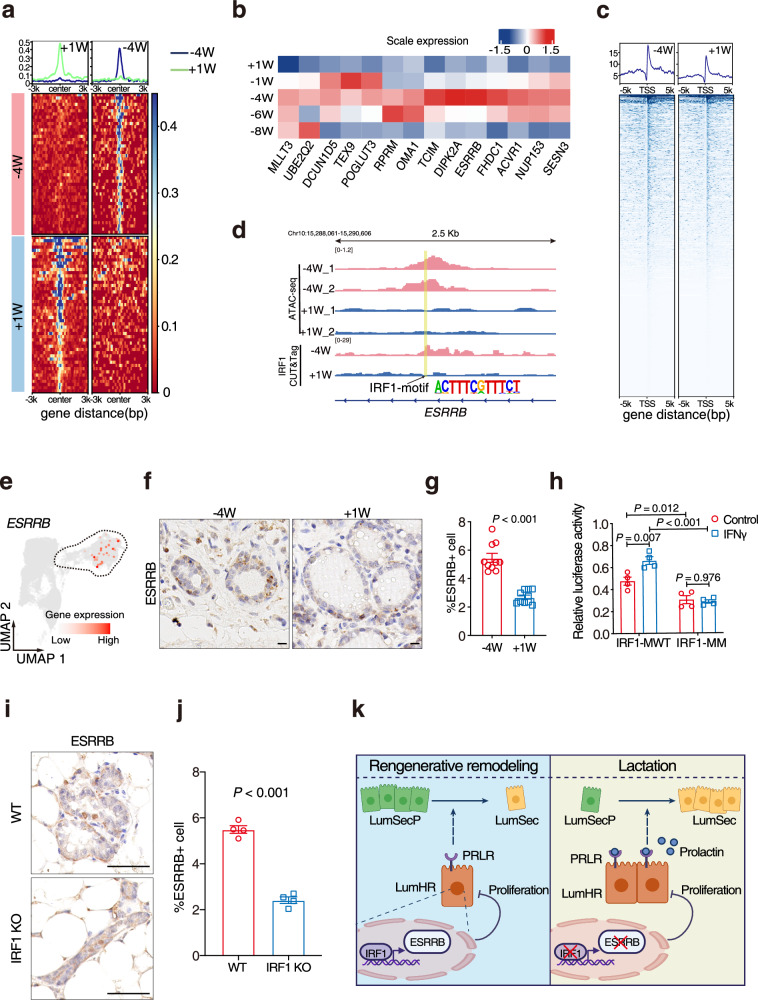
Identification of the target gene of IRF1 in LumHR cells. **a** The genomic loci with IRF1 motifs are selected and the ATAC-seq signal intensity is shown in heatmaps. The average signal intensity is shown on top. **b** Heatmap displaying the transcriptional level of genes presumably bound by IRF1. *n* = 3 goats per group. **c** Heatmaps showing the signal intensity of IRF1 CUT&Tag in goat mammary tissues at −4W and +1 W. The average signal intensity is shown on top. **d** ATAC-seq and IRF1 CUT&Tag profiles at the *ESRRB* locus in −4W and +1 W are shown. The differential regions between −4W and +1 W with IRF1 motifs are highlighted in yellow. **e** UMAP plot showing the specific expression of *ESRRB* in LumHR cells by scRNA-seq data. **f**, **g** Immunohistochemical staining and quantification of ESRRB in goat mammary tissues at −4W and +1 W. Representative images of Immunohistochemical staining (**f**). Nuclei are counterstained with hematoxylin. *n* = 10 sections from 5 goats per group. Scale bars, 20 μm. Two-sided Student’s *t*-test. **h** Luciferase reporter assays in goat mammary epithelial cells. Cells are transfected with WT IRF1 motif (IRF1-MWT) or IRF1-motif site mutation (IRF1-MM) vector and treated with IFNγ or not. *n* = 4 biological replicates. Two-way ANOVA test. **i**, **j** Immunohistochemical staining and quantification of ESRRB in mouse WT or IRF1-KO mammary tissues under RR. Representative images of Immunohistochemical staining (**i**). Nuclei are counterstained with hematoxylin. *n* = 4 mice per group. Scale bars, 50 μm. Two-sided Student’s *t*-test. **k** The proposed model in the current study is that a reduction of LumHR cells triggered by IRF1-ESRRB signaling upregulation promotes the accumulation of LumSecP during RR in ruminants. LumHR cells control the differentiation of LumSecP to LumSec cells through the PRLR pathway and regulate the cell composition of luminal lineages during RR. Created with BioRender.com.

To identify the potential targets of IRF1 in LumHR cells, we identified genes associated with open chromatin regions that were enriched with IRF1 motifs at −4W (Fig. 6a, b). Among these candidates, estrogen-related receptor beta (*ESSRB*), myeloid/lymphoid or mixed-lineage leukemia translocated to chromosome 3 protein (*MLLT3*), transcriptional and immune response regulator (*TCIM*), and sestrin 3 (*SESN3*) were RR-associated, transcriptionally upregulated, and linked to increased accessible chromatin regions at −4W (Fig. 1d; Supplementary Dataset S2). To determine the targets of IRF1 in LumHR cells, we integrated the ATAC-seq and IRF1 CUT&Tag assays on goat mammary tissues collected at −4W and +1 W (Fig. 6c; Supplementary Tables S1 and S5). Our analysis showed that the chromatin accessibility signals at IRF1-binding motifs in the *ESRRB* and *MLLT3* loci were markedly elevated at −4W compared to +1 W, whereas such changes were not observed for *TCIM* and *SESN3* (Fig. 6d; Supplementary Fig. S16a). Importantly, *ESRRB* was the only candidate gene specifically expressed in LumHR cells (Fig. 6e; Supplementary Fig. S16b). Analyses of a published IRF1 ChIP-seq dataset derived from mouse mammary cells further confirmed that *ESRRB* is a conserved target of IRF1 (Supplementary Fig. S17a)^58^.

Combined with the fact that ESRRB functions as a cell proliferation repressor^59–63^, it is likely that IRF1 inhibits the proliferation of LumHR cells by targeting *ESRRB*. Therefore, we examined the expression of *ESRRB* in goat mammary tissues (Supplementary Fig. S17b). Immunohistochemical analysis confirmed a significant increase of ESRRB at −4W within inner luminal cells (Fig. 6f, g). Two IRF1 motifs were found at the second intron of *ESRRB* as revealed by ATAC-seq and CUT&Tag, highlighting that IRF1 may regulate the expression of *ESRRB* through this *cis*-regulatory element (Chr10: 15,289,041-15,289,115, pink marked in Supplementary Fig. S17c). To investigate whether IRF1 directly controls *ESRRB* expression through the *cis*-regulatory element identified, we synthesized and constructed fragments of either the wild type (IRF1-MWT) or a version with mutated IRF1 motifs (IRF1-MM) into a luciferase reporter vector (Supplementary Fig. S17c). The reporter assays revealed that mutation of the IRF1 binding motifs significantly diminished the transcriptional activity of the *cis*-regulatory element (Fig. 6h). Meanwhile, the transcriptional activities of the IRF1-MWT were enhanced by IFNγ treatment (final concentration at 100 units/mL) but blocked in the mutated version (Fig. 6h). In addition, the expression of the ESRRB protein was suppressed significantly in the mammary gland of IRF1-KO mice compared with WT during RR and at 9 weeks after birth (Fig. 6i, j; Supplementary Fig. S17d). We concluded that *ERSSB* is a previously unidentified IRF1 target in LumHR cells.

## Discussion

A typical involution of the mammary gland is a degenerative process characterized by massive cell death, leading to complete loss of alveolar structures^14,64^. Compared with the typical involution, RR is characterized by the renewal of alveolar structures essential for the subsequent lactation. Here, our data uncovered that the RR involves three main phases in dairy goats. The first phase initiates right after the weaning and is characterized by cell death in luminal cells, leading to a notable reduction in lumen size and cell number over a period of approximately 2–3 weeks. During the second phase, the degenerated lobuloalveolar structures are maintained, while the LumSecP are generated and progressively accumulated. Simultaneously, a reduction in the number of LumHR cells is observed, accompanied by the suppression of proliferation. The third phase initiates the renewal of the mammary gland, characterized by a substantial expansion of LumHR cells, the maturation of secretory cells, and the restoration of lumen size. This phase, extending to the early lactation, is essential for the functional restoration of the mammary glands and prepares for the subsequent lactation upon hormone stimulation. In this work, we have unveiled a genetic program that governs the dynamic changes occurring at the morphological, cellular, and transcriptional levels during RR.

We first report a single-cell resolution atlas of goat mammary tissues, which includes 7 major cell types and 14 distinct cell states. While we utilized a few conventional markers for cell type identification in this atlas, we have also identified numerous marker genes that have not been widely reported before. This atlas serves as a valuable resource for future studies. Within the luminal cells, our data have identified four cell states with diverse biological functions and these cell proportions undergo dynamic changes during RR. Specifically, we have observed that the accumulation of LumSecP responds to rapid mammary gland functional restoration upon the load of pregnancy hormones. However, it is important to note that the expansion of LumSecP poses a risk of tumorigenesis, as they can give rise to triple-negative breast cancer^22,65–67^. Therefore, the tight control of LumSecP over-accumulation is critical during RR. In this study, our findings indicate that it is crucial to have a low proportion of LumHR cells at −4W to prevent the pre-maturation of LumSecP ahead of parturition (Fig. 6k). This finding highlights the critical function of LumHR cells in tightly controlling the expansion of LumSecP during the regenerative state.

Prolactin is a key hormone that initiates alveologenesis. It acts directly on luminal cells to stimulate milk synthesis via signal transducer and activator of transcription 5 (STAT5) activation. The importance of PRLR-STAT5 signaling for lactation is highlighted by studies of female *Prlr* or *Stat5* knockout mice, which develop severe defects of mammary alveologenesis and lactation^15,68^. In this study, we discovered that the *PRLR* is predominantly expressed in LumHR cells. This is consistent with previous data in mice, which showed that phosphorylated STAT5 is localized in ER- and PR-positive luminal cells, and responds to prolactin^69,70^. Prolactin promotes the differentiation of LumSecP in mammary organoids, possibly through paracrine signaling or direct cell–cell contact. This implies that LumHR cells may serve as a bridge to connect upstream signals and the subsequent cell response, ensuring a more precise regulation at the organ level. This finding suggests an under-appreciated spatial distinction in prolactin signaling in the mammary gland, and advances our understanding of the role of prolactin-activated LumHR cells in tightly controlling the maturation of LumSec cells during RR.

LumHR cells are suggested to function in converting endocrine reproductive cues into paracrine or autocrine factors (e.g., AREG^71^, RANKL^72,73^, and WNT4^37^) that are able to orchestrate functional differentiation of the mammary lineages. However, previous reports detailing the identity and mechanisms of regulating the LumHR cells remain scarce^74^. Specifically, upregulation of the transcription factor IRF1 at −4W blocks the expansion of LumHR cells, while loss of function of IRF1 induces abnormal proliferation of luminal cells. A few studies reported a role of IRF1 in inhibiting the growth of breast cells; however, IRF1 was also shown to suppress the premature epithelial apoptosis at the first phase of typical involution in mice^51,52,54^. Such discrepancy suggested that the function of IRF1 in the mammary gland may be modulated by the local microenvironment of normal mammary tissues, such as hormones^49,51^. Although IFNγ is a well-established cytokine that activates IRF1 signaling, it is acknowledged that IFNγ may exert additional effects on mammary organoids beyond IRF1 induction. Importantly, the observations that the abnormal proliferation of LumHR cells and activation of the ER pathway in IRF1-KO mice underscore a novel role of IRF1 associated with estrogen-dependent activities in tuning the ratio of LumHR cells. This argument is further supported by the fact that *ESRRB*, a novel target of IRF1, suppresses estrogen-dependent cellular functions such as cell proliferation^59–63^. These findings highlight that the downregulation of IRF1-ESRRB signaling, leading to a reduction in LumHR cells, promotes gland regeneration during RR (Fig. 6k). Although gene KO goats are better for dissecting in vivo mechanisms, our findings demonstrate that goat mammary organoids provided a convenient way to validate the function of LumHR cells involved in RR. However, the upstream events that activate the IRF1-ESRRB pathway in LumHR cells remain unclear. Given that immune cells (e.g., macrophages and T cells) are recruited to clean up apoptotic luminal cells during involution and are known to produce interferons^1,75–77^, it is possible that immune cell-derived interferons activate IRF1 in LumHR cells, thereby regulating the accumulation of LumSecP during RR (Fig. 6k). Further studies are warranted to establish a link between immune cells and luminal cells during the remodeling of the mammary gland.

We recognize that longitudinal transcriptomic comparisons alone cannot unequivocally distinguish remodeling-associated expression changes from those specific to pregnancy or typical involution. Nonetheless, future studies using cohorts undergoing pregnancy without involution and involution without concurrent pregnancy will be helpful for fully disentangling remodeling-specific transcriptional features. A further caveat is that our scRNA-seq analysis revealed only a modest increase in LumSec cells after remodeling, which appears inconsistent with the schematic model (Fig. 6k). We attribute this discrepancy largely to technical limitations. LumSec cells at the lactation stage are often enriched with large lipid droplets and display an “adipocyte-like” phenotype, making them particularly prone to loss during centrifugation and inefficiently capture by single-cell sequencing platforms. Despite this underrepresentation, the scRNA-seq dataset still showed a significant increase in the LumSec cells at +1 W compared with −4W within luminal lineages. Importantly, immunofluorescence analyses provided complementary evidence for the expansion of LumSec cells (FABP3+ ) in goat mammary tissues after remodeling.

Overall, our study advances our understanding of the regulatory mechanisms underlying the dynamic changes in luminal lineage composition during RR in ruminants and possibly other mammals as well. The discovery of LumHR-dependent regulation of tissue remodeling provides insights into developing new strategies for intervening the mammary gland renewal to accelerate milk production in dairy animals.

## Materials and methods

### Ethics declarations

The animal study underwent a review process and received approval from the Experimental Animal Management Committee of Zhejiang University (Approval Number: ZJU20230204). Moreover, all experimental procedures adhered to the National and Institutional Guidelines for the Ethical Use of Experimental Animals in Research (GBT35825-2018 and GBT35892-2018).

### Animals and tissue collection

In this study, 3-year-old goats in their second lactation were selected for tissue collection. All goats were at a similar lactation stage (210 ± 3 days) and body weight (59 ± 1.5 kg). Mating was performed around day 210 of lactation. The mammary tissues from the dairy goats were collected by surgery at −8W, −6W, −4W, −1W, and +1W to cover the entire RR process for paraformaldehyde fixation and bulk RNA-seq. The mammary tissues from dairy goats at -4W and +1W were also collected for scRNA-seq, ATAC-seq and CUT&Tag. Each period of RR was collected from at least three individuals. For each individual used for bulk or single-cell RNA-seq, mammary tissue was collected from 2 or 3 areas of one udder to make a sample pool (about 3 g). To mitigate the potential impact of multiple biopsies on our observations, we carefully selected biopsy sites across different udders to ensure representative sampling while minimizing tissue damage. Goats without pregnancy were selected to induce the typical involution after weaning. The mammary tissues of goats (*n* = 3) for the typical involution (without a linked pregnancy) were collected at 2 weeks, 4 weeks, and 9 weeks after weaning, corresponding to −6W, –4W, and +1W during RR. B-ultrasound was employed to determine the pregnancy status of each goat. Twenty-two goats were used in this study and the basic information about the goats used for tissue collection and sequencing is described in Supplementary Table S1.

### Mouse strains and experimental design

IRF1 KO (strain #: 002762) and *ROSA-DTA* (strain #: 009669) mice were obtained from Jackson Laboratories^57^. *H11-CAG-LSL-ZsGreen* mice (strain #: NM-KI-200319) were purchased from Shanghai Model Organisms Center, Inc. Animals were maintained in a C57BL/6 genetic background. Animals were housed in specific pathogen-free conditions at 22 °C, with a humidity of 50%–60% and a 12-h light-dark cycle. Allocation to each group was determined by the animal’s genotype. The mammary tissues of WT or IRF1 KO mice were collected at 9 weeks (end of puberty) for whole-mount staining, H&E, immunofluorescence, immunohistochemistry staining, and bulk RNA-seq. To induce typical involution, the lactating mice were weaned at lactation day 14. The mammary tissues of typical involution were collected on day 8 after forced weaning for whole-mount staining. To induce an RR status consistent with that in goats, the lactating mice cohabited with males on day 10 of lactation, and pups were removed at lactation day 14 to induce mammary involution as described previously^18^. The mammary tissues of RR were collected on day 8 after forced weaning for H&E, immunofluorescence, and immunohistochemistry staining.

### Histology, immunofluorescence, and immunohistochemistry imaging

Paraffin-embedded tissues were sectioned and subjected to and histochemical staining, while OCT-embedded tissues or organoids were sectioned for immunofluorescence staining. For histochemical staining, sections were incubated three times with 3% H_2_O_2_ for 10 min at room temperature to inactivate endogenous peroxidases, and then blocked with 10% duck serum for 2 h and incubated with primary antibodies at 4 °C overnight. The sections were incubated with secondary antibodies for 1 h at room temperature. For immunofluorescence staining, sections were blocked for 2 h, and then incubated with primary antibodies at 4 °C overnight and secondary antibodies for 1 h at room temperature. For immunofluorescence staining, goat mammary tissues were stained with primary antibodies against ALDH1A3 (#25167-1-AP, Proteintech), FABP3 (#10676-1-AP, Proteintech), KRT17 (#17516-1-AP, Proteintech), KRT14 (#SC65-06, HUABIO), KRT18 (conjugated with iFluor™ 488, #SZ80-07, HUABIO), KRT8 (#10384-1-AP, ProteinTech), PR (#25871-1-AP, ProteinTech), ER (#21244-1-AP, ProteinTech), and PCNA (#ab29, Abcam). Goat mammary organoids were stained with ALDH1A3 (#25167-1-AP, Proteintech), FABP3 (#10676-1-AP, Proteintech), and KRT18 (conjugated with iFluor™488, #SZ80-07, HUABIO). Mouse mammary tissues (WT, IRF1 KO, H11-CAG-LSL-ZsGreen, and ROSA-DTA) were stained with ALDH1A3 (#25167-1-AP, Proteintech), FABP3 (#10676-1-AP, Proteintech), PCNA (#ab29, Abcam) and KRT18 (conjugated with iFluor™488, #SZ80-07, HUABIO). Secondary antibodies were conjugated with CoraLite 488 (#SA00013-2, ProteinTech) or CoraLite 594 (#SA00013-4, ProteinTech). The organoids were stained with V5-tagged anti-PRLP produced by Ankyron (EG40356), in combination with Anti-V5 Fluorotag conjugated to Alphalux 488 (VK11A, Ankyron). For immunohistochemistry, goat mammary tissues were stained with IRF1 (homemade anti-goat rabbit polyclonal antibody) and ESRRB (#22644-1-AP, ProteinTech). Mouse mammary tissues were stained with PR (#ab101688, Abcam), ER (#ab32063, Abcam), β-casein (sc-166530, Santa Cruz), and ESRRB (#22644-1-AP, ProteinTech), combined with peroxidase kit (#PK-4001, Vectorlabs, USA). Fixed mammary glands from IRF1 KO, WT, and ROSA-DTA mice were processed for whole-mount staining with carmine alum (#C6152, Sigma, USA) following established protocols^78^. Immunofluorescence-stained specimens were imaged using a confocal microscope (LSM880, Zeiss, Germany), while immunohistochemistry slides were scanned with a digital slide scanner (Pannoramic 250 Flash III, 3DHISTECH, Hungary). The Image J software (http://imagej.nih.gov/ij/) was used to measure the lumen sizes and the cell number.

### Goat mammary organoid cultures and treatments

The goat mammary organoids were obtained and cultured following our recent protocol^47^. Briefly, primary mammary organoids were prepared from mammary tissue of 8-month-old female dairy goats without mating. The fresh mammary tissue was minced into fragments and digested with 1*×* collagenase/hyaluronidase (10*×*, 3000 U/mL collagenase and 1000 U/mL hyaluronidase, 07912, StemCell Technologies, Cambridge, USA). The digestion mixture was incubated at 37 °C for 1.5 h with gentle shaking. Erythrocytes were lysed with 0.8% ammonium chloride for 5 min, followed by treatment with 20 U/mL DNase I for 5 min at room temperature. The suspension underwent three rounds of differential centrifugation to remove single cells and lymphocytes. The organoids were resuspended and kept on ice for 3D culture. The primary mammary organoids were mixed with growth factor reduced Matrigel (#354230, Corning, USA) and plated in domes in a 24-well culture plate (one dome per well, 100 mL of Matrigel per dome).

For prolactin treatment, the organoids were incubated with prolactin (2 μM, #CW72, Novoprotein, China) on day 5 and collected for bulk RNA-seq, scRNA-seq, immunofluorescence and immunohistochemistry staining on day 8. For the IFNγ treatment, the mammary organoids were incubated with a culture medium containing IFNγ (100 units/mL, recombinant human IFNγ, #CM40, Novoprotein) or PBS from day 2 to day 9. The growth area of the organoids was acquired using a microscope (Nikon, Japan) on days 1, 3, 5, 7, and 9. The quantification of growth area images was carried out using Image J software by transforming the figures into 8-bit and measuring the area covered by the organoids. The organoids were collected on day 9 for the EdU staining. The EdU (EdU-Click 647, Sigma, USA) was incubated with the culture medium about 2 h before the collection of the organoids for staining. The lipid droplets in the organoids were stained with BODIPY (#790389, Thermo Fisher Scientific, USA) according to the manufacturer’s procedure. The stained organoids were digitally photographed using a confocal microscope (LSM 880, Zeiss).

### Bulk RNA-seq and data analysis

Goat or mouse mammary tissues and mammary organoids were collected to purify total RNA using poly-T oligo-attached magnetic beads. The raw sequence data were processed using standard Illumina pipelines for base-calling and fasta file generation (Supplementary Table S2). Genes that possessed less than five raw reads in half of the samples, mitochondrial genes, and ribosomal genes were removed. Paired-end reads were mapped to the primary assembly of the Saanen dairy goat genome (ASM4283598v1). A gene was considered to be expressed in a sample if its count value was equal to or greater than 1 in that sample. Genes with a count value of zero across all samples were eliminated. Differential expression analysis was conducted using the DESeq2 R package. Genes with an adjusted *P* value < 0.05 and log_2_|fold change| > 1 identified by DESeq2 were classified as differentially expressed. The genes were analyzed using GO or GSEA to identify enriched pathways and biological processes. GO enrichment and GSEA analysis were performed using the Metascape (https://metascape.org) or clusterProfiler R package. The raw data of bulk RNA-seq from goat mammary tissues and organoids were uploaded to the National Center for Biotechnology Information Sequence Read Archive (SRA) database under the accession numbers PRJNA922362 and PRJNA994875, respectively.

### Generation of single-cell suspensions

Single-cell experiments were carried out on goat mammary tissue samples (*n* = 3) at −4W and +1W, respectively. The samples were finely minced and then incubated in a digestion solution consisting of 1*×* Collagenase/Hyaluronidase (Stemcell Technology, #07912), EpiCult-B (#05611, Stemcell Technology, USA), and 5% FBS at 37 °C in a shaking incubator for two hours. The dissociated cells were then subjected to red blood cell lysis, followed by a 5-min treatment with 1 U/mL dispase (#07913, Stem Cell Technology) and 0.1 mg/mL DNase (#07470, Stemcell Technology), and a 5-min treatment with Trypsin-EDTA (#07901, Stemcell Technology), before being filtered through a 40 mm cell strainer. Finally, the cells were suspended in PBS containing 0.3% BSA for scRNA-seq. Mammary organoids were collected and incubated in ice-cold recovery solution for 45 min to facilitate matrix dissolution. The recovered organoids were then dissociated into single-cell suspensions using Trypsin-EDTA, followed by resuspension in PBS containing 0.3% BSA for scRNA-seq.

### Single-cell library construction and sequencing

The preparation of single-cell RNA libraries and subsequent sequencing were carried out using the 10*×* Chromium 3′ library construction kit v3 (10*×* Genomics, Pleasanton, CA, USA) following the manufacturer’s instructions for single-cell capture and cDNA library generation (Supplementary Table S3). The libraries were then sequenced on an Illumina NovaSeq sequencer at Novogene Technology Co., Ltd (Tianjin, China). The raw data of scRNA-seq from mammary tissue and organoids were uploaded to the National Center for Biotechnology Information SRA database under the accession numbers PRJNA922365 and PRJNA1322110, respectively.

### Processing and quality control of scRNA-seq

The paired-end reads generated by the Illumina NovaSeq were processed and aligned to the goat genome (ASM4283598v1) using the Cell Ranger 7.1.0 software from 10*×* Genomics. To ensure high-quality data, we applied rigorous filters to exclude cells with UMI counts lower than 1000 or higher than 60,000, gene counts below 500 or above 2500, and mitochondrial gene ratios exceeding 10%^22^. The filtered data were subsequently analyzed using Seurat v5.0.1, and potential cell doublets were identified using DoubletCollection. Finally, the samples were merged and normalized using Harmony (v1.1.0) to avoid sampling bias.

### scRNA-seq data processing

The variable genes were determined using the “FindVariableGenes” function in Seurat with default parameters. Clusters were identified via the “FindCluster” function (at a resolution of 0.3) and visualized using UMAP. Marker genes for each cluster were determined with the Wilcoxon rank-sum test using the “FindAllMarkers” function (at a logFC threshold of 0.5) implemented in Seurat. We used “DoHeatmap” to visualize markers of each cell type. To assess the function of each cell type, we used the “GSEA” function in the clusterProfiler (v4.0.5) R package to perform functional enrichment of these markers. CytoTRACE (v0.3.3) was used to predict the differentiation potential of luminal cells^79^. Monocle 2 and Monocle 3R packages were used to discover cell state transitions^80^. The subsequent pseudotime trajectory analysis was performed for all luminal cell subtypes. We also used SCENIC (v1.2.4) to identify regulon activity and cell-specific regulons in scRNA-seq dataset of goat mammary tissue. The public scRNA-seq data of mouse mammary glands were extracted and downloaded from the Gene Expression Omnibus (GEO) database under accession code GSE106273^41^. The one-to-one conserved genes were used for the cross-species analysis between goat and mice. The regulatory networks were constructed based on the TF modules and visualized with a heatmap. Pathway analysis was performed on DEGs using MSigDB v7.0.

### Cell sorting

The cells used in the ATAC-seq analysis were the same dissociated cells from goat mammary tissues used in the scRNA-seq. The cells were permeabilized with Triton X-100 (0.55 pmol/cell) for 5 min^81^, and then incubated with a KRT18 antibody (#ET1603-8, HUABIO). The secondary antibody is conjugated with Alexa Fluor^@^647 (#ab150079, Abcam). Cell sorting and analysis were conducted on a BD FACSAria II cell sorter and FlowJo. The sorted cells were collected for ATAC-seq.

### ATAC-seq and data processing

The ATAC-seq procedure was performed following a published protocol^82^, with two biological replicates. Library preparations were carried out at Novogene Technology Co., Ltd (Tianjin, China) and sequenced using an Illumina Hiseq platform (Supplementary Table S4). The ATAC-seq reads were aligned to Saanen dairy goat genome (ASM4283598v1) using BWA MEM v0.7.17 with standard parameters. Duplicated reads were marked using Picard MarkDuplicates v2.19.0, and the alignment was filtered using Samtools v1.9 to remove multi-mapping and duplicated reads. Peaks were called from the filtered and sorted BAM files using MACS2 v2.1.2 with standard parameters^83^. Consensus peaks were then obtained by merging peak calls. Heat maps and metaplots were generated using deepTools version 3.1.2. Finally, DNA sequence motif analysis of ATAC-seq peaks was performed using HOMER^84^. The raw data of ATAC-seq was uploaded to the National Center for Biotechnology Information SRA database under the accession number PRJNA923168.

### CUT-Tag library preparation and sequencing

The CUT&Tag was performed using a commercial kit following the manufacturer’s protocol (N259-YH01, Novoprotein, China). For IRF1 CUT&Tag, mammary tissues were harvested at −4W and +1W. Tissues were homogenized to isolate nuclei, from which approximately 50,000 nuclei per sample were collected and bound to Concanavalin A-coated magnetic beads. Bead-bound nuclei were then incubated overnight at 4 °C with IRF1 rabbit antibody (1:100 dilution, 11335-1-AP, Proteintech). Tagmented DNA was purified and subjected to sequencing on the Illumina NovaSeq platform (Novogene Technology Co., Ltd, Tianjin, China). Bioinformatic analysis was conducted as previously described^85^. Raw reads were filtered and mapped to the goat reference genome (ASM4283598v1) using Bowtie2 (v2.4.1). The raw data from the IRF1 CUT&Tag were uploaded to the National Center for Biotechnology Information SRA database under the accession number PRJNA1259229.

### Vector construction and virus production

The pAAV-Cre expression vector was obtained from VectorBuilder (Guangzhou, China). For virus packaging, the serotype 9 packaging plasmid and the adenoviral helper plasmid were sourced from Addgene (#112865 and #112867). A 3-kb mouse *Prlr* promoter (Chr15:10,175,244-10,178,244) was cloned according to ATAC-seq and H3K4me3 ChIP-seq^58^. The *Prlr* promoter was amplified from mouse genomic DNA and cloned upstream of the Cre coding sequence in the pAAV-Cre plasmid, generating the *pAAV-pPrlr-Cre* construct. The resulting AAV vectors carried either Cre recombinase or a null transgene (*pAAV-Control*) under the control of the *Prlr* promoter. AAV particles were produced and purified through cesium chloride gradient centrifugation. Viral titers were quantified using a densitometric dot-blot assay, yielding final titers of 1.3 × 10^13^ genome copies (GC)/mL.

### Intraductal injection

Intraductal injections were carried out as previously described^86^. For *H11-CAG-LSL-ZsGreen* reporter mice, the mammary glands were harvested 7 days post-injection and processed for immunofluorescence analysis. For functional studies, *ROSA-DTA*^+/*−*^ mice undergoing RR were intraductally injected with *AAV-pPrlr-Cre* or *AAV-Control* (approximately 1.3 × 10^11^ GC per gland) ten days prior to the expected delivery date. To assess the effects of LumHR cell ablation, mammary glands were collected on lactation day 2 for qPCR, H&E, wholemount, immunofluorescence, and immunohistochemistry staining.

### RNA extraction and PCR

Total RNA was extracted from goat and mouse mammary tissues using TRIzol reagent (#15596026, Life Technologies, USA) and reverse-transcribed into cDNA with the PrimeScript™ RT Reagent Kit (TaKaRa, Japan). Subsequently, RT-qPCR was performed on a StepOnePlus Real-Time PCR System (Applied Biosystems, USA) using a SYBR Green PCR Master Mix (#A46110, Applied Biosystems). Primers were as described in Supplementary Table S6. *GAPDH* used as an internal control both in goats and mice. The comparative ΔCT method was used for data analysis.

### Luciferase analysis

The fragments of the WT IRF1 motif (IRF1-MWT, containing site –1713 to +65) and the IRF1-motif site mutation (IRF1-MM) were constructed into the pGL3-basic vector. For the luciferase assay, primary goat mammary epithelial cells cultured in 48-well plates at 80%–90% confluence were co-transfected with 300 ng of the vector plus 10 ng of the control vector (Renilla luciferase) per well using Lipofectamine 2000 reagent (Thermo Fisher Scientific). The cells were incubated with recombinant dog IFNγ (Novoprotein, #CM40) at 100 units/mL or control (PBS) after 24 h of initial culture, and then harvested at 48 h. The dual-Luciferase Reporter assay kit (#E1910, Promega, USA) was used to measure luciferase activity on a Fluoroskan Ascent apparatus (#2805880, Thermo Fisher Scientific). The relative luciferase activity was calculated as the ratio of firefly luciferase compared with renilla luciferase activity.

### Statistical analysis

The relative ratio of each cell type was determined by dividing the cell number of a specific cell type by the total cell number in the scRNA-seq dataset. For all the staining, more than five visual fields with different directions in each section were randomly selected for statistics. Sample sizes, statistical tests, and *P* values are indicated in the respective figure or figure legend. The number of junctions, branches, ductal length and ductal tips, the diameter of the lumen, mammary tree filled and alveoli number per mm^2^ in the tissue were quantified using Image J software as described previously^87,88^. Given that the nuclear localization of PR and ER is necessary for their transcriptional role, we only counted cells with positive nuclei for the quantification of anti-PR+ and anti-ER+ luminal cells in immunofluorescence staining of mammary tissues. The mean fluorescence intensity for BODIPY staining in organoids was carried out using Image J software. The mean fluorescence intensity was normalized to the number of nuclei per organoid. All experiments were performed to have at least three biological replicates to ensure power for statistical analysis. Statistical significance was defined as *P* < 0.05.

## Supplementary information

Supplementary Information

Supplementary Datasets

## Data Availability

All sequencing data are available in the National Center for Biotechnology Information Sequence Read Archive (PRJNA994875, PRJNA923168, PRJNA922365, PRJNA1259229, PRJNA922362, and PRJNA1322110). All data are available within the main text or the supplementary materials, or available from the corresponding authors upon reasonable request.
