# Supplementary material for: Luminal hormone-responsive cells tune the regenerative remodeling of mammary glands in large mammals
Source: Cell Discov. 2025 Dec 30;11:105. doi: 10.1038/s41421-025-00848-3 (PMC12749961; doi:10.1038/s41421-025-00848-3)
Supplement: Supplementary file 1 — Supplementary Information [file 41421_2025_848_MOESM1_ESM.pdf]

Supplementary Information for

Luminal hormone-responsive cells tune the regenerative remodeling of mammary glands in large mammals

Yongtao Li[1,†], Liping Zhang[2,†], Tao Luo[1,†], Wenying Zhang[1], Teng Wang[3], Fanming Liu[4], Shengda Lin[4], Jun Luo[5], Jianxin Liu[1], Jinrong Peng[1], Chaochen Wang[3,*], Wei Wang[2*] and Hengbo Shi[1,6,*]

This file includes:

Supplementary Tables S1 to S6
Supplementary Figure S1 to S17 and figure legends
Legends for Supplementary Datasets 1 to 9

Other supporting materials for this manuscript include the following:

Supplementary Datasets 1 to 9

Table S1. Basic information applied to sequencing samples of goat mammary tissues.

Sample	Goat	Age	State	Lactation	Pregnant	Sequencing methods
-8W_1	Goat_1	3 years	-8W	No	Yes	Bulk-seq
-8W_2	Goat_2	3 years	-8W	No	Yes	Bulk-seq
-8W_3	Goat_3	3 years	-8W	No	Yes	Bulk-seq
-6W_1	Goat_1	3 years	-6W	No	Yes	Bulk-seq
-6W_2	Goat_2	3 years	-6W	No	Yes	Bulk-seq
-6W_3	Goat_3	3 years	-6W	No	Yes	Bulk-seq
-4W_1	Goat_1	3 years	-4W	No	Yes	Bulk-seq
-4W_2	Goat_2	3 years	-4W	No	Yes	Bulk-seq
-4W_3	Goat_3	3 years	-4W	No	Yes	Bulk-seq
-4W_4	Goat_4	3 years	-4W	No	Yes	Bulk-seq
-4W_5	Goat_5	3 years	-4W	No	Yes	Bulk-seq
-4W_6	Goat_6	3 years	-4W	No	Yes	Bulk-seq
-1W_1	Goat_1	3 years	-1W	No	Yes	Bulk-seq
-1W_2	Goat_2	3 years	-1W	No	Yes	Bulk-seq
-1W_3	Goat_3	3 years	-1W	No	Yes	Bulk-seq
+1W_1	Goat_1	3 years	+1W	Yes	No	Bulk-seq
+1W_2	Goat_2	3 years	+1W	Yes	No	Bulk-seq
+1W_3	Goat_3	3 years	+1W	Yes	No	Bulk-seq
+1W_4	Goat_7	3 years	+1W	Yes	No	Bulk-seq
+1W_5	Goat_7	3 years	+1W	Yes	No	Bulk-seq
+1W_6	Goat_9	3 years	+1W	Yes	No	Bulk-seq
-4W_7	Goat_10	3 years	-4W	No	Yes	scRNA-seq
-4W_8	Goat_11	3 years	-4W	No	Yes	scRNA-seq
-4W_9	Goat_12	3 years	-4W	No	Yes	scRNA-seq

+1W_7	Goat_10	3 years	+1W	Yes	No	scRNA-seq
+1W_8	Goat_11	3 years	+1W	Yes	No	scRNA-seq
+1W_9	Goat_12	3 years	+1W	Yes	No	scRNA-seq
-4W_1 _ATAC	Goat_10	3 years	-4W	No	Yes	ATAC-seq
-4W_2 _ATAC	Goat_11	3 years	-4W	No	Yes	ATAC-seq
+1W_1 _ATAC	Goat_10	3 years	+1W	Yes	No	ATAC-seq
+1W_2 _ATAC	Goat_11	3 years	+1W	Yes	No	ATAC-seq
-4W_1 CUT& Tag	Goat_10	3 years	-4W	No	Yes	CUT&Tag
-4W_2 CUT& Tag	Goat_11	3 years	-4W	No	Yes	CUT&Tag
+1W_1 CUT& Tag	Goat_12	3 years old	-4W	Yes	No	CUT&Tag
+1W_1 CUT& Tag	Goat_13	3 years	-4W	Yes	No	CUT&Tag

Table S2. Quality control information for bulk RNA-sequencing data of mammary tissue

Sample	Clean reads	Q20 in Raw read	Error rate (%)
-8W_1	44,713,418	97.46%	0.03%
-8W_2	51,159,246	97.42%	0.03%
-8W_3	43,673,260	97.61 %	0.03%
-6W_1	42,716,646	97.33%	0.03%
-6W_2	53,055,314	96.99%	0.03%
-6W_3	50,931,094	97.37%	0.03%
-4W_1	44,197,480	96.98%	0.03%
-4W_2	42,152,388	97.69%	0.03%
-4W_3	46,696,942	97.22%	0.03%
-4W_4	43,257,320	97.51%	0.03%
-4W_5	41,629,592	97.38%	0.03%
-4W_6	46,148,598	97.72%	0.03%
-1W_1	44,637,348	97.47%	0.03%
-1W_2	49,114,720	97.33%	0.03%
-1W_3	51,810,196	97.04%	0.03%
+1W_1	40,358,114	97.42%	0.03%
+1W_2	50,333,866	97.26%	0.03%
+1W_3	44,236,590	97.67%	0.03%
+1W_4	45,594,290	97.25%	0.03%
+1W_5	43,614,626	97.97%	0.03%
+1W_6	42,424,632	97.71%	0.03%

Table S3. Quality control information for scRNA sequencing data of goat mammary tissue and organoids.

Sample	Raw reads	Q30 in Raw read	Number of Cells	Mean Reads per Cell	Median Genes per Cell	Q30 Bases in RNA Read	Q30 Bases in UMI
Tissue -4W_1	509,005,416	91.07%	6,375	79,844	1,021	91.1%	95.6%
Tissue -4W_2	519,814,542	91.24%	6,091	94,462	811	91.4%	95.6%
Tissue -4W_3	575,368,817	91.42%	8,484	61,270	954	91.2%	95.7%
Tissue +1W_1	574,134,353	90.78%	4,313	123,325	1,144	89.2%	94.5%
Tissue +1W_2	531,901,032	89.21%	7,895	69,801	988	89.9%	94.6%
Tissue +1W_3	551,081,801	89.90%	10,569	54,322	853	90.8%	94.7%
Organoids (Control)	396,306,217	96.66%	10,151	39,041	2,901	96.7%	97.3%
Organoids (Prolactin)	387,727,125	96.74%	7,727	50,178	3,188	96.8%	97.5%

Table S4. Quality control information for ATAC-seq data.

Sample	Raw reads	Q30 in Raw read	Count of narrow peak	FRiP	Count of summits
-4W_1_ATAC	44, 830,244	91.43%	78,316	28.6110%	95096
-4W_2_ATAC	43,076,400	91.33%	35,321	15.1675%	40323
+1W_1_ATAC	43,532,735	90.66%	57,127	22.1271%	72455
+1W_2_ATAC	45,823,434	90.41%	60,210	23.4974%	76951

Table S5. Quality control information for IRF1 CUT&Tag.

Sample	Raw reads	Q30 in Raw read	Error rate (%)
-4W_1_IRF1_CUT&Tag	52,620,468	91.43%	0.02%
-4W_2_IRF1_CUT&Tag	56,022,234	91.33%	0.02%
+1W_1_IRF1_CUT&Tag	47,091,140	90.66%	0.02%
+1W_2_IRF1_CUT&Tag	46,517,202	90.41%	0.02%

Table S6. Sequences of primers using in qPCR for goat and mouse (ms) tissue.

Gene	Sequence (5' to 3')
Goat IRF1	GGATGCCTGTCTGTTTCGGA<br>TTGGGATCCGGCTCCTTTTC
Goat GAPDH	GCAAGTTCCACGGCACAG<br>CTTCAAGTGAGCCCCAGCC
Goat SOX9	GCAAGCTCTGGAGACTGCTG<br>GGCCGTTCTTCACCGACTTC
Goat RUNX1	ACTCTGCCCATCGCTTTCAA<br>CTTCCACTCCGACCGACAAA
Goat WNT4	GCCGGCAGGAAGGCCA<br>CACGTCTTTACCTCGCAGGA
Mouse Aldh1a3	ATCAACAACGACTGGCACGA<br>CCTTGTCCACATCGGGCTTA
Mouse Areg	CTGTTGCTGCTGGTCTTA<br>AGTAGTCGTAGTCCCCTGT
Mouse Cd36	TGTGGAGCAACTGGTGGATG<br>CGTGGCCCGGTTCTAATTCA
Mouse Csn3	ACTGTGGCCAATCCTGAAGC<br>GTTGAAATTTGGTTCCAGACCTTT
Mouse Elf5	GTGGCATCAAGAGTCAAGACTGTC<br>CTCAGCTTCTCGTACGTCATCCTG
Mouse Fabp3ABP3	CAGGTGGCTAGCATGACCAA<br>CAGCGTCACCAGTGACTTGA
Mouse Foxa1	GGATCCCCGCTACTCCTTTA<br>AGCACGGGTCTGGAATACAC
Mouse Foxp1	CTCACTGCCTGTAGTGCCTC<br>GTCACGTCTCACCCCATTCA
Mouse Gapdh	AGGTCGGTGTGAACGGATTTG<br>TGTAGACCATGTAGTTGAGGTCA
Mouse LALBA	TGAATGGGCCTGTGTTTTAT<br>CACGCTATGTCATCATCCAA
Mouse Runx1	CCTTCAGGAGAGGTGCGTTT<br>CTCGTGCTGGCATCTCTCAT
Mouse S100a8	GTCCTCAGTTTGTGCAGAATATAAA<br>GCCAGAAGCTCTGCTACTCC
Mouse S100a9	TTGGCAACCTTTATGAAGAAAGAGA<br>GTGGGTTGTTCTCATGCAGC
Mouse Wap	CTGGAGCATTCTATCTTCA

TGCCTCATCAGCCTAGTTCT

Fig. S1 Identification of the genes and pathways associated with morphological changes during regenerative remodeling. (a) H&E staining in goat mammary tissues after weaning at 2 weeks, 4 weeks, and 9 weeks in typical involution (n = 5 goats per time point), which are relative to -6W, -4W, and +1W in regenerative involution, respectively. Scale bar, 50 μm. (b) Immunofluorescence staining and measurement of lumen cell number in goat mammary tissues at -8W, -4W and +1W. Immunofluorescence staining was conducted on the goat mammary glands, with KRT18 labeling the luminal cells in green and DAPI labeling the nuclei in blue. Images at -8W, -4W, and +1W were taken, with quantification of KRT18-positive cells presented (*n* = 90 lumens per group). An ANOVA analysis was performed for the cell number in the lumen. Scale bar, 40 μm. (c) Volcano plots depicting the differentially expressed genes (DEGs) during RR at various time points (relative to +1 week), with a threshold at log_2Foldchange > 1 and $P < 0.05$. (d) A bar plot showing the number of differentially expressed genes at -8W, -6W, -4W, and -1W compared to +1W by bulk RNA-seq. (e) GSEA snapshots of function enrichment analysis (-4W vs. +1W). The terms specific to mammary gland development are displayed.

Fig. S2 ATAC-seq analysis for KRT18-positive luminal cells in goat mammary tissue. (a) Flow sorting of goat mammary luminal cells using anti-KRT18 at -4W and +1W. (b) A heatmap visualizing the Pearson correlation of read counts of ATAC-seq among the four samples from the flow sorting. (c) Pie charts depicting the distribution of genomic features among the top 1,000 peaks at -4W and +1W. (d) A bar plot showcasing the representative GO terms enriched by the top 1000 peak genes at -4W and +1W. (e) A heatmap displaying the expression of 69 known regulators associated with cell proliferation across all time points. Four milk protein synthesis-related genes are displayed as control (labeled in red). (f) Relative expression levels of selected genes from (e) involved in cell proliferation in the goat mammary tissues at -4W and +1W were measured by qPCR (*n* = 3 goats per group).

Fig. S3 Identification of the cell types by scRNA-seq in goat mammary gland in RR. (a) Flowchart overview of single-cell RNA-seq of the goat mammary tissues (Created with BioRender.com). (b) Heatmap displaying the marker-gene signatures of all cell types. (c) Dot plot showing the expression of representative marker genes for each cell type. (d-e) UMAP plots illustrating the representative marker genes of luminal, basal, immune, fibroblast, and pericyte cells in the goat mammary gland. *AREG* and *NFIB* are known genes involved in mammary gland development while *STAT5* is critical for milk synthesis. The color key ranges from gray to red representing low to high gene expression levels. (f) Immunofluorescence-stained mammary tissue for anti-KRT14 (basal cells) in red, anti-KRT8 (luminal cells) and anti-KRT17(basal cells) in green, and DAPI in blue. Scale bar, 50 μm.

Fig. S4 Gene signatures of the four identified luminal cell types. (a) UMAP plots showing the expression of selected marker genes related to milk protein synthesis (*CSN1S1*, *CSN1S2*, *LGB*), milk fat (*CD36*, *BTN1A1*, *LPL*), and mammary gland development (*GATA3*, *ELF5*, STAT5A, *STAT5B*, *TNFSF11,* and *STAT3*) in the four luminal subtypes. (b) Violin plots showing the expression of four milk protein genes in each luminal cell types. (c) CytoTRACE values for the four luminal cell types at -4W and +1W. (d) Heatmap illustrating the expression of signature genes of luminal subtypes. GO terms enriched in the signature genes were presented by a bar plot.

Fig. S5 Lineage analysis for luminal cells in RR. (a) Pseudotemporal cell ordering of all luminal subtypes along differentiation trajectories. (b) Heatmap showing the expression level of each ordered gene in LumSecP and LumSec cells. (c) Representative enriched GO terms in ordering genes from different modules in (b). Different colors represent different gene modules. (d) Expression of indicated genes along pseudotime in LumSecP and LumSec cells differentiation.

Fig. S6 Enrichment of differentially expressed genes across RR in luminal cell types. (a) Enrichment analysis of RR-associated program across all cell types in scRNA-seq dataset of goat mammary gland. The average gene expression over the cells within each cluster was calculated and used for the plot. The top 3 enriched cell types are highlighted in red. (b) Enrichment of RR-associated program across all cell types at -4W and +1W, respectively. The average gene expression over the cells within each cluster was compared between -4W and +1W using the Wilcoxon test. The P-value for each cell type is displayed. (c) Among the RR-associated program, 20 and 10 genes were specifically detected in LumHR cells and LumSecP, respectively. A heatmap displaying the expression of these specific genes in LumHR cells and LumSecP across all time points. (d) Representative enriched GO terms for the genes specifically expressed in LumHR cells. (e) Representative enriched GO terms for the genes specifically expressed in LumSecP.

Fig. S7 Establishment of goat mammary organoids. (a) Schematic for the establishment of goat mammary organoids and experimental treatment with prolactin. (b) Brightfield image of goat mammary organoid culture. Scale bar, 200 μm. (c) Immunofluorescence stained goat mammary organoids for anti-KRT17 (basal cells) and anti-KRT18 (luminal cells) in green, and DAPI in blue. Scale bar, 50 μm. (d) Representative immunofluorescence images of goat mammary organoids treated without (control) or with prolactin. Milk fat (green, Bodipy), cytoskeleton (red, phalloidin), and nuclei (blue, DAPI) were stained in the goat mammary organoids. Scale bar, 50 μm. (e) Quantifcation of BODIPY staining mean intensity per organoid normalized to the number of nuclei per organoid. Each dot corresponds to one dome of organoids stimulated with or without prolactin (n = 9 per group). The data are normalized to prolactin group. (f) GSEA analysis was performed using the 69 known regulators as a gene set to assess the proliferation index of mammary organoids treated by prolactin or not. GSEA graphical output for the enrichment of the gene set

is displayed. (g) Heatmaps displaying the expression of 69 known regulators in goat mammary organoids treated by prolactin or not (control group). n = 3 biological replicates per group.

Fig.S8 Identification of luminal cell types by scRNA-seq in goat mammary organoids. (a) UMAP plot showing identified basal and luminal cell types within goat mammary organoids treated with or without prolactin. Cells are annotated and color-coded by type. Subsets of cell types, including basal, LumSec, LumHR, LumSecP and LumProg, are labeled. n = 10,820 cells. (b) UMAP plot displaying the basal and luminal cells identified in organoids treated with (Prolactin) or without prolactin (Control). (c) Dot plot showing the expression of representative marker genes for each cell type. (d) Proportions of luminal cell types in goat mammary organoids for both Control and Prolactin groups. (e) UMAP plots showing the expression of selected marker genes in four luminal subtypes. (f) Violin plots showing the expression of PRLR genes in LumHR cells in Control and Prolactin groups. (g) Immunofluorescence staining of PRLR in goat mammary organoids incubated without prolactin. Scale bar, 10 μm.

Fig.S9 Cross-species comparison of scRNA datasets between goat and mouse mammary glands. (a) UMAP plot displaying the identified basal and luminal cell types within the mouse mammary gland. The publicly available single-cell transcriptome datasets from mouse mammary tissues are sourced from Bach et al., 2017. The mouse mammary cells represent the involution, gestation, and lactation stages. (b) UMAP plots showing the expression of general lineage marker genes for basal (Krt14) and luminal (Krt18) subtypes. (c) Dot plot showing the expression of representative marker genes for luminal cell types in both goat and mouse mammary glands. (d) Heatmap depicting the similarity between goat and mouse luminal cell types. The goat mammary cell types are indicated by "g" while mouse mammary cell

types are indicated by "m". (e) UMAP plots displaying the expression of four marker genes specific to LumHR cells in mice, which are also specific to LumHR cells in goats. (f) Representative image showing co-staining of PR (LumHR marker) and KRT18 in mouse mammary tissues on lactation day 0. Scale bar, 20 μm

Fig. S10 Effects of LumHR cells ablation through AAV intraductal injection. (a) H&E staining in mouse mammary tissues under regenerative remodeling (RR) and typical involution. To induce an RR status consistent with that in goats, the lactating mice cohabited with males on day 10 of lactation, and pups were removed at lactation day 14 to induce mammary involution. Scale bar, 50 μm. (b) Illustration of full *Prlr* promoter revealed by ATAC-seq and H3K4me3 ChIP-seq datasets of mouse mammary gland. A 3,000 bp fragment corresponding to the full-length mouse *Prlr* promoter (Chr15:10,175,244-10,178,244, marked in yellow) was amplified from mouse genomic DNA and cloned upstream of the Cre coding sequence in the pAAV-Cre plasmid, generating the pAAV-pPrlr-Cre construct. (c) Experimental setup used in AAV-pPrlr-Cre intraductally injected lactation mammary gland of heterozygous *H11-CAG-LSL-ZsGreen* mice. (d) Immunofluorescence stained lactation mammary tissue of H11 mice intraductally injected with AAV-pPrlr-Cre for PR and GFP and DAPI in blue. Scale bar, 100 μm. (e) H&E staining for mammary glands from *ROSA-DTA*$^{+/-}$ mice (lactation day 2) intraductally injected with AAV-pPrlr-Cre or AAV-Control. (f) Alveoli quantification (per mm^2) in (e). n = 3 mice per group. Scale bars, 50 μm. (g) The qPCR analysis for luminal differentiation markers (*Foxp1*, *Areg*, *Elf5*, *Wap*, *Csn3*, *Fabp3*, *Foxa1* and *Cd36*) and progenitor-associated genes (*Aldh1a3*, *S100a8* and *S100a9*) in AAV-pPrlr-Cre and AAV-Control mice. n = 3 mice per groups.

Fig. S11 SCENIC analysis showing the regulon activity of IRF1 in LumHR cells in goat mammary tissue. (a) Dot plot displaying top cluster-specific regulons in each

luminal subtype. The size of the dot represents regulon activity. The cluster-specific regulons were analyzed using the SCENIC software. RSS = regulon specificity score. (b) UMAP plots showcasing the expression of *IRF1* in luminal cell types. The color key ranging from gray to red represents gene expression levels that range from low to high. (c) A network plot visualizing the predicted target genes regulated by IRF1 in LumHR cells. Red nodes represent regulators. Blue nodes represent target genes while yellow nodes represent differential expression genes in LumHR cells at -4W compared with +1W. (d) Relative expression levels of *IRF1* in the goat mammary tissues at -4W and +1W were measured by qPCR.. n = 3 goats per group.

Fig. S12 SCENIC analysis showing the regulon activity of IRF1 in mouse mammary glands. (a) Network plot showing the transcription factor regulatory network in mouse luminal subtypes. Pink nodes indicate regulators with a number of target genes. The node of Irf1 is marked in red. (b) Heatmap showing the active transcription factors clustered by hormone-sensing differentiation (Hsd) and hormone-sensing progenitors (Hsp) in typical involution of mouse mammary gland through SCENIC analysis. IRF1 is activated in differentiated hormone-sensing cells.

Fig. S13 Morphological phenotypes in IRF1-KO mouse mammary glands. (a) Representative carmine-stained mouse mammary gland whole mounts at day 2 and day 5 of wild type (WT, n = 4 mice) and IRF1-KO mice (n = 3 mice) during typical involution of mammary glands. Scale bar, 0.1mm. The pups were removed at lactation on day 14 to induce mammary involution. The mammary tissues of WT and IRF1-KO mice were collected on day 2 and day 5 after forced weaning was initiated. (b) Brightfield images showing the organoids incubated with IFNγ or not. Scale bar, 200 μm. (c) Bar plot showing the relative size of goat mammary organoids (n = 15 dome per group). A two-sided Student's t-test was performed. (d-e) EdU staining and quantification in goat mammary organoids treated with IFNγ or not (control).

Representative images of EdU staining. Scale bar, 50μm. The data shown are presented as mean ± SEM (*n* = 12 domes per group). Two-sided Student's t-test. (f) Representative images of carmine-stained mammary gland whole mounts in WT (*n* = 7 mice) and IRF1-KO (*n* = 3 mice) mice at 9 weeks. Scale bar, 0.2 mm. (g and h) Quantification of distance from the branch tips to lymph node (g) and epithelial filled fat pad (h) in WT or IRF1-KO mammary tissues of mice in (f). Data are mean ± SEM. Two-sided Student's t-test. (i) Representative images of H&E staining of typical and regenerative involution of mammary gland in WT (*n* = 4 mice) and IRF1-KO (*n* = 3 mice) of mice. Scale bar, 100 μm.

Fig. S14 Proliferation of luminal cells in IRF1-KO mice. (a-b) Immunofluorescence staining (a) and quantification (b) of PCNA in mammary tissues from 9-week-old wild-type (WT) or IRF1-KO mice. The total number of inner luminal cells (enclosed in while dotted line) was counted using Image J software. The data are presented as mean ± SEM. *n* = 9 sections from 3 mice per group. Two-sided Student's t-test. Scale bar, 50 μm. (c) Representative enriched GSEA terms in mammary glands of WT (upregulated and marked in red) and IRF1-KO (downregulated and marked in blue) mice. *n* = 3 mice per group. (d) Preranked GSEA graphical output for the enrichment in IRF1-KO mice mammary glands of the gene sets from the Molecular Signatures Database Hallmarks collection.

Fig. S15 Displaying of known PR and ER response genes in ChIP database. (a) Mouse Genome browser tracks of ER signals on five ER response gene loci referring to Fig. 4q. (b) Mouse Genome browser tracks of PR signals on five PR response gene loci referring to Fig. 4q.

Fig. S16 Displaying the expression of IRF1 downstream candidates. (a) ATAC-seq and IRF1 CUT&Tag profiles at the *MLLT3, TCIM* and *SESN3* locus in

-4W and +1W are shown. The IRF motif regions are highlighted in yellow. (b) UMAP plots showcasing the expression of *MLLT3, TCIM* and *SESN3* in luminal cell types. The color key ranging from gray to red represents gene expression levels that range from low to high.

Fig. S17 Identification of *ERSSB* as an IRF1 target in LumHR cells. (a) Mouse *ESRRB* intergenic regions in which peaks with IRF1-binding motifs were on average more accessible in breast cancer cells (AT-3) compared to macrophages. IFNγ enhanced the accessibility in breast cancer cells (AT-3). (b) UMAP plot showing the specific expression of *ESRRB* in LumHR cells by scRNA dataset of goats. (c) The DNA sequence of the predicted IRF1-binding region at the *ESRRB* locus is shown. Predicted IRF1 binding motifs are highlighted in red, which are deleted in the mutant. (d) Representative immunohistochemical staining of ESRRB in mammary tissues from WT or IRF1 KO mice at 9 weeks. ESRRB protein was almost completely suppressed in the mammary glands of IRF1-KO mice at 9 weeks. Nuclei were counterstained with hematoxylin. Scale bar, 50 μm.

Fig. S1

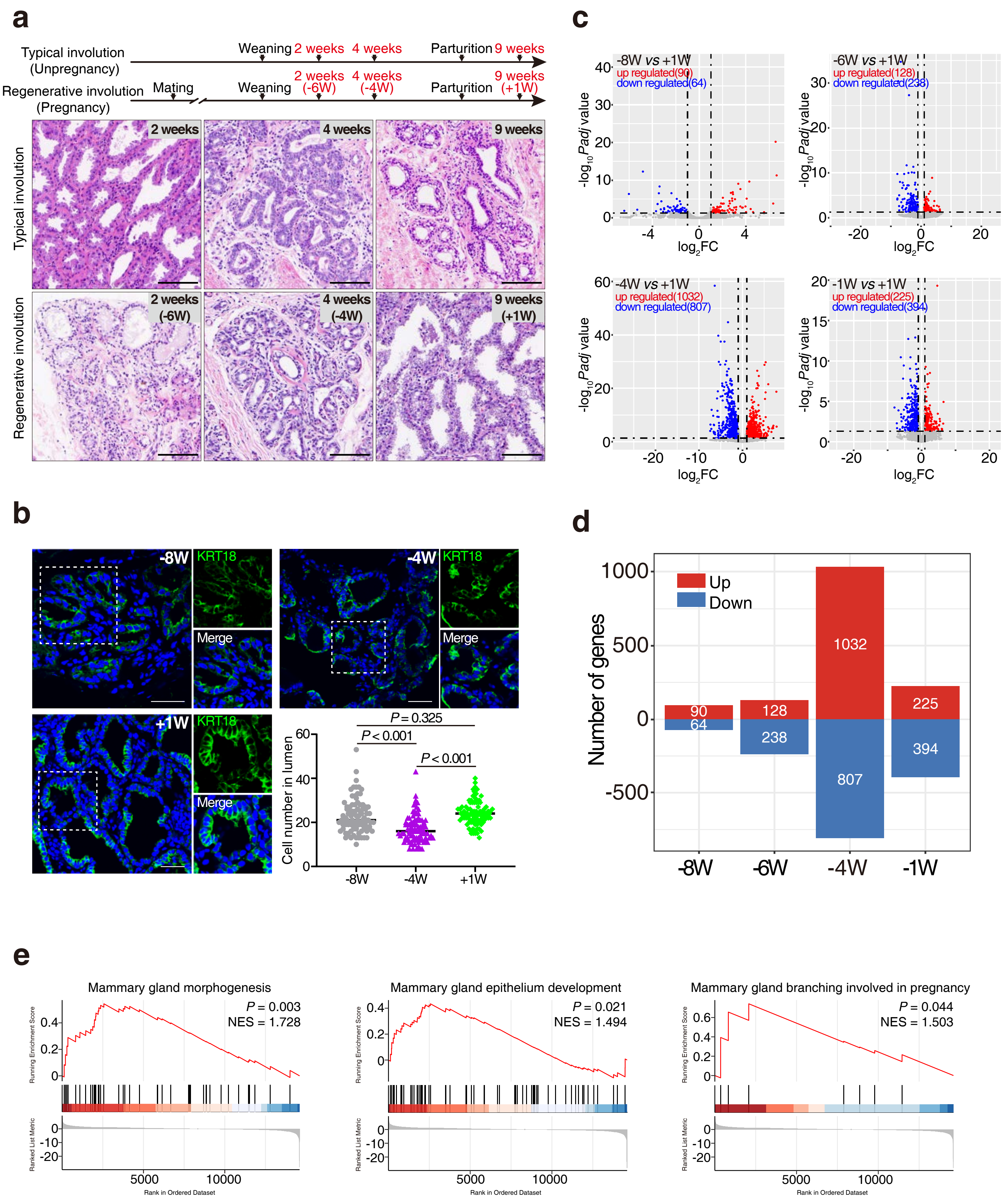

a
Typical involution (Unpregnancy)
Regenerative involution (Pregnancy)
Mating
Weaning
2 weeks
4 weeks
Parturition
9 weeks
(-6W)
(-4W)
(+1W)
Typical involution
Regenerative involution
c
-8W vs +1W
up regulated(90)
down regulated(64)
-6W vs +1W
up regulated(128)
down regulated(238)
-4W vs +1W
up regulated(1032)
down regulated(807)
-1W vs +1W
up regulated(225)
down regulated(394)
$-\log_{10}$Padj value
$\log_2$FC
b
-8W
-4W
+1W
KRT18
Merge
P = 0.325
P < 0.001
P < 0.001
Cell number in lumen
d
Up
Down
Number of genes
90
64
128
238
1032
807
225
394
-8W
-6W
-4W
-1W
e
Mammary gland morphogenesis
P = 0.003
NES = 1.728
Mammary gland epithelium development
P = 0.021
NES = 1.494
Mammary gland branching involved in pregnancy
P = 0.044
NES = 1.503
Running Enrichment Score
Ranked List Metric
Rank in Ordered Dataset

Fig. S2

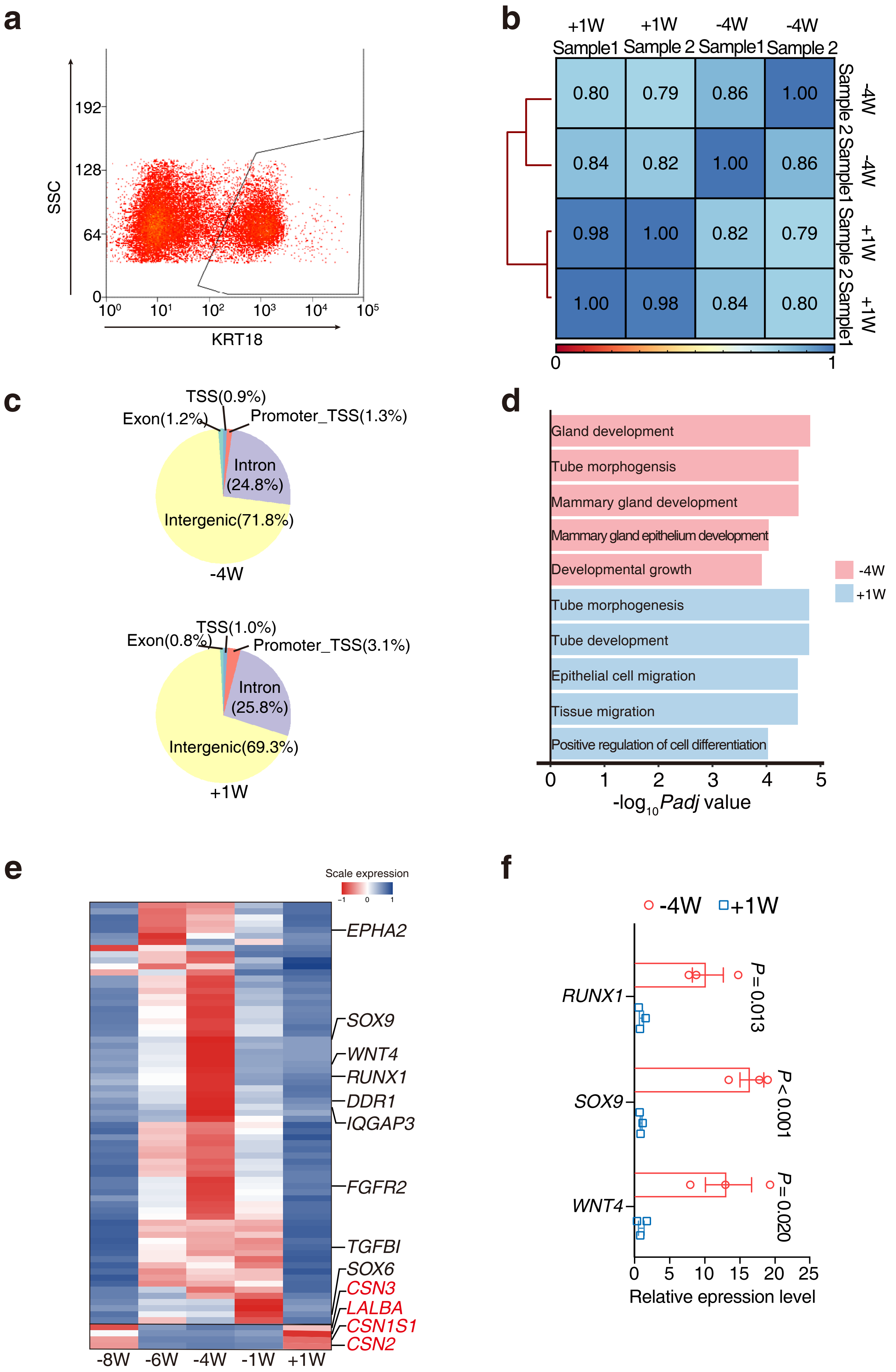

a
SSC
192
128
64
0
KRT18
b
+1W Sample1
+1W Sample 2
-4W Sample1
-4W Sample 2
0.80 0.79 0.86 1.00
0.84 0.82 1.00 0.86
0.98 1.00 0.82 0.79
1.00 0.98 0.84 0.80
0
1
c
TSS(0.9%)
Exon(1.2%)
Promoter_TSS(1.3%)
Intron (24.8%)
Intergenic(71.8%)
-4W
TSS(1.0%)
Exon(0.8%)
Promoter_TSS(3.1%)
Intron (25.8%)
Intergenic(69.3%)
+1W
d
Gland development
Tube morphogensis
Mammary gland development
Mammary gland epithelium development
Developmental growth
Tube morphogenesis
Tube development
Epithelial cell migration
Tissue migration
Positive regulation of cell differentiation
-4W
+1W
0 1 2 3 4 5
-log10Padj value
e
Scale expression
EPHA2
SOX9
WNT4
RUNX1
DDR1
IQGAP3
FGFR2
TGFBI
SOX6
CSN3
LALBA
CSN1S1
CSN2
-8W -6W -4W -1W +1W
f
-4W +1W
RUNX1
SOX9
WNT4
P = 0.013
P < 0.001
P = 0.020
0 5 10 15 20 25
Relative epression level

Fig. S3

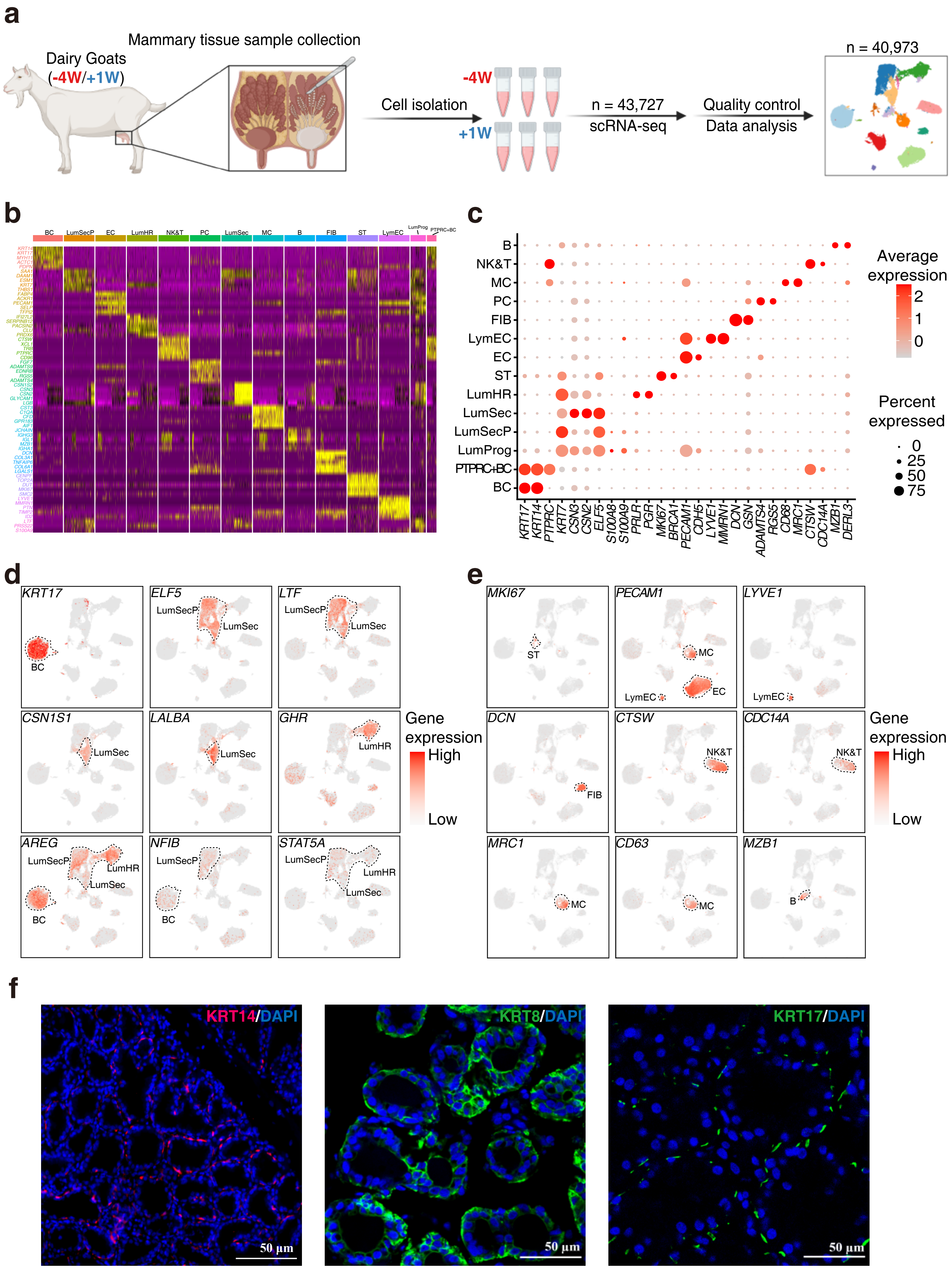
a
Mammary tissue sample collection
Dairy Goats
(-4W/+1W)
Cell isolation
-4W
+1W
n = 43,727
scRNA-seq
Quality control
Data analysis
n = 40,973
b
c
Average expression
Percent expressed
d
e
Gene expression
High
Low
f
KRT14/DAPI
KRT8/DAPI
KRT17/DAPI
50 μm

Fig. S4

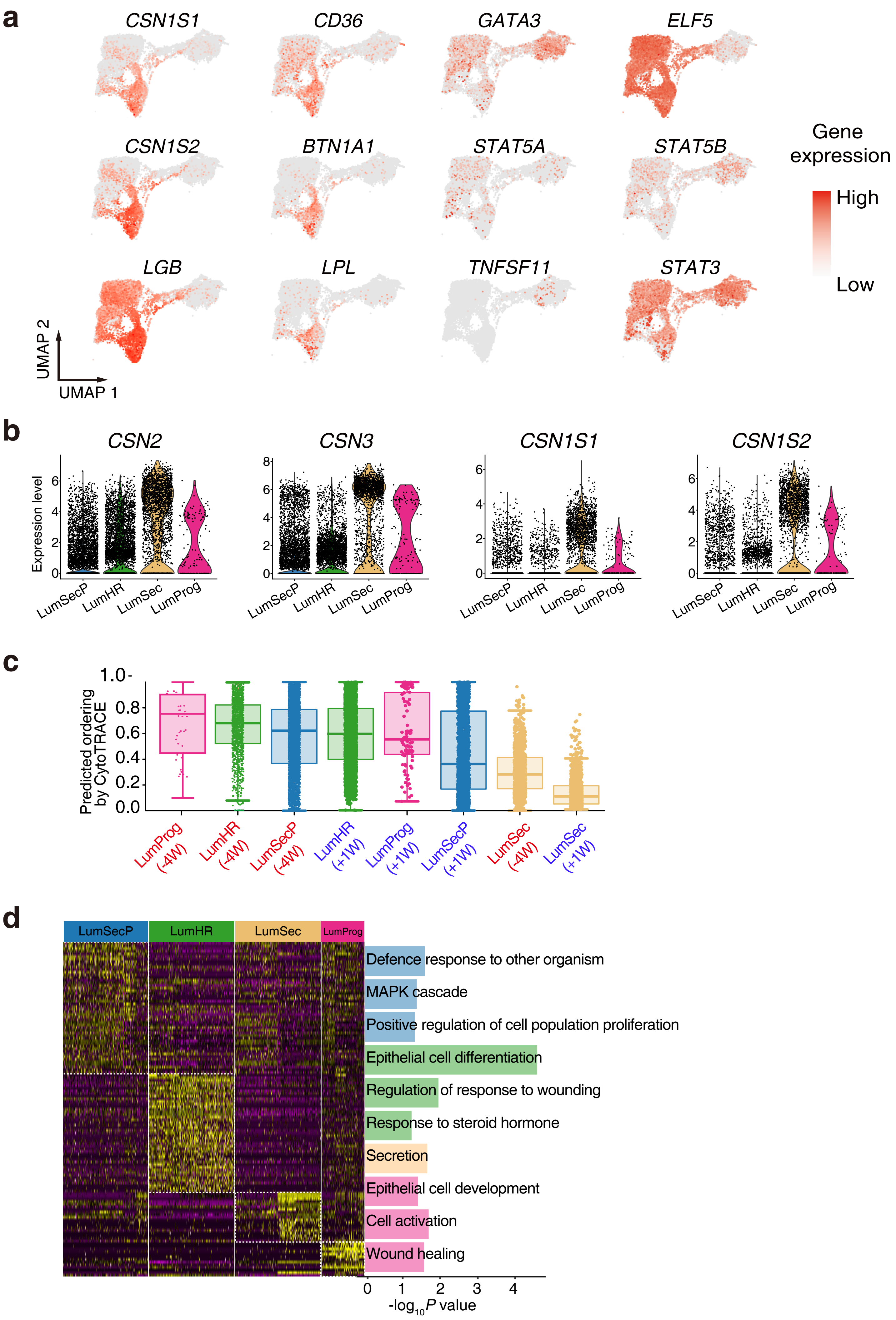
a
CSN1S1
CD36
GATA3
ELF5
CSN1S2
BTN1A1
STAT5A
STAT5B
LGB
LPL
TNFSF11
STAT3
Gene expression
High
Low
UMAP 2
UMAP 1
b
CSN2
CSN3
CSN1S1
CSN1S2
Expression level
LumSecP
LumHR
LumSec
LumProg
c
Predicted ordering by CytoTRACE
LumProg (-4W)
LumHR (-4W)
LumSecP (-4W)
LumHR (+1W)
LumProg (+1W)
LumSecP (+1W)
LumSec (-4W)
LumSec (+1W)
d
LumSecP
LumHR
LumSec
LumProg
Defence response to other organism
MAPK cascade
Positive regulation of cell population proliferation
Epithelial cell differentiation
Regulation of response to wounding
Response to steroid hormone
Secretion
Epithelial cell development
Cell activation
Wound healing
-log10P value

Fig. S5

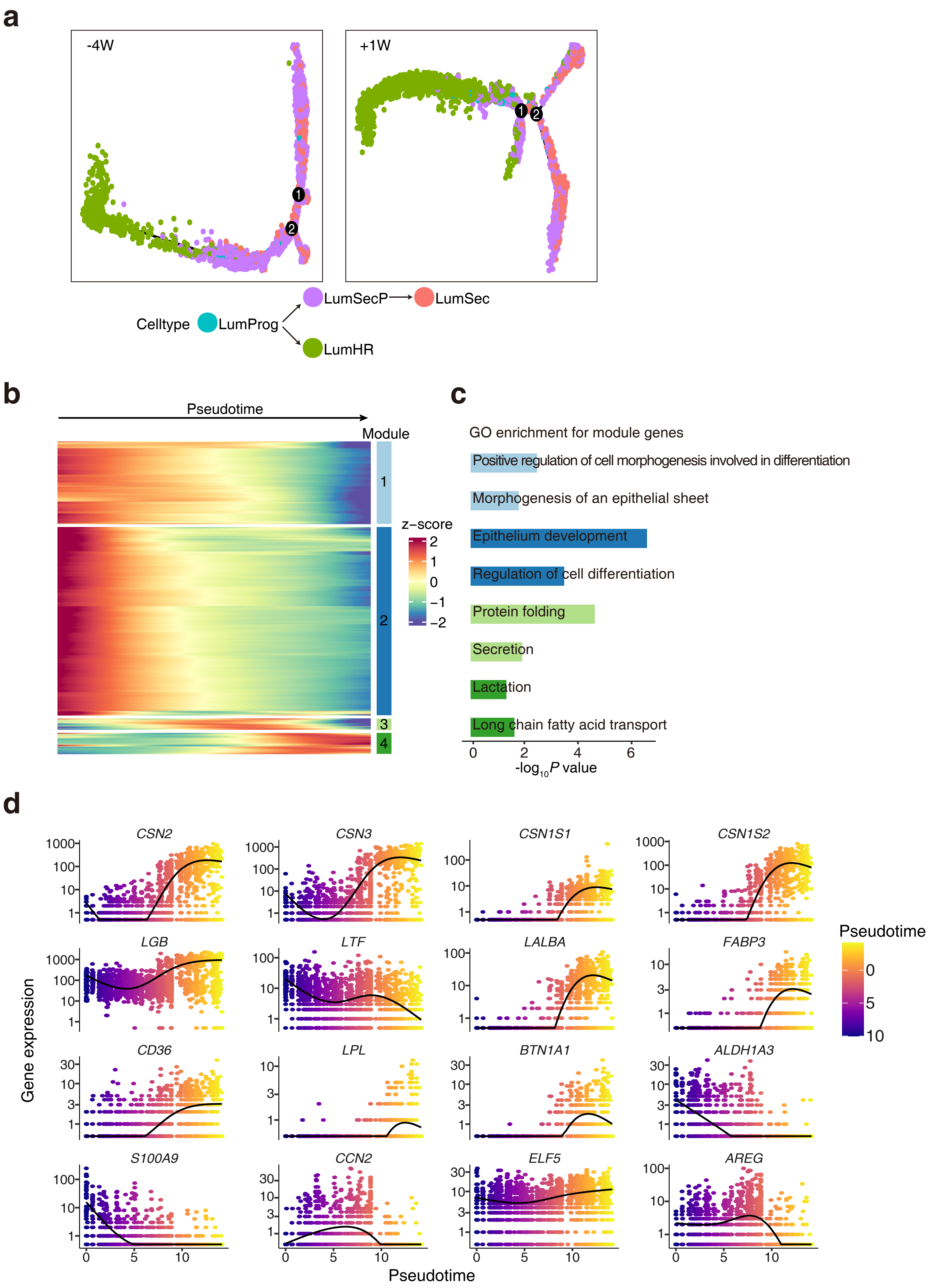
a
-4W
+1W
Celltype
LumProg
LumSecP
LumSec
LumHR
b
Pseudotime
Module
1
2
3
4
z−score
2
1
0
−1
−2
c
GO enrichment for module genes
Positive regulation of cell morphogenesis involved in differentiation
Morphogenesis of an epithelial sheet
Epithelium development
Regulation of cell differentiation
Protein folding
Secretion
Lactation
Long chain fatty acid transport
-log10P value
d
CSN2
CSN3
CSN1S1
CSN1S2
LGB
LTF
LALBA
FABP3
CD36
LPL
BTN1A1
ALDH1A3
S100A9
CCN2
ELF5
AREG
Gene expression
Pseudotime

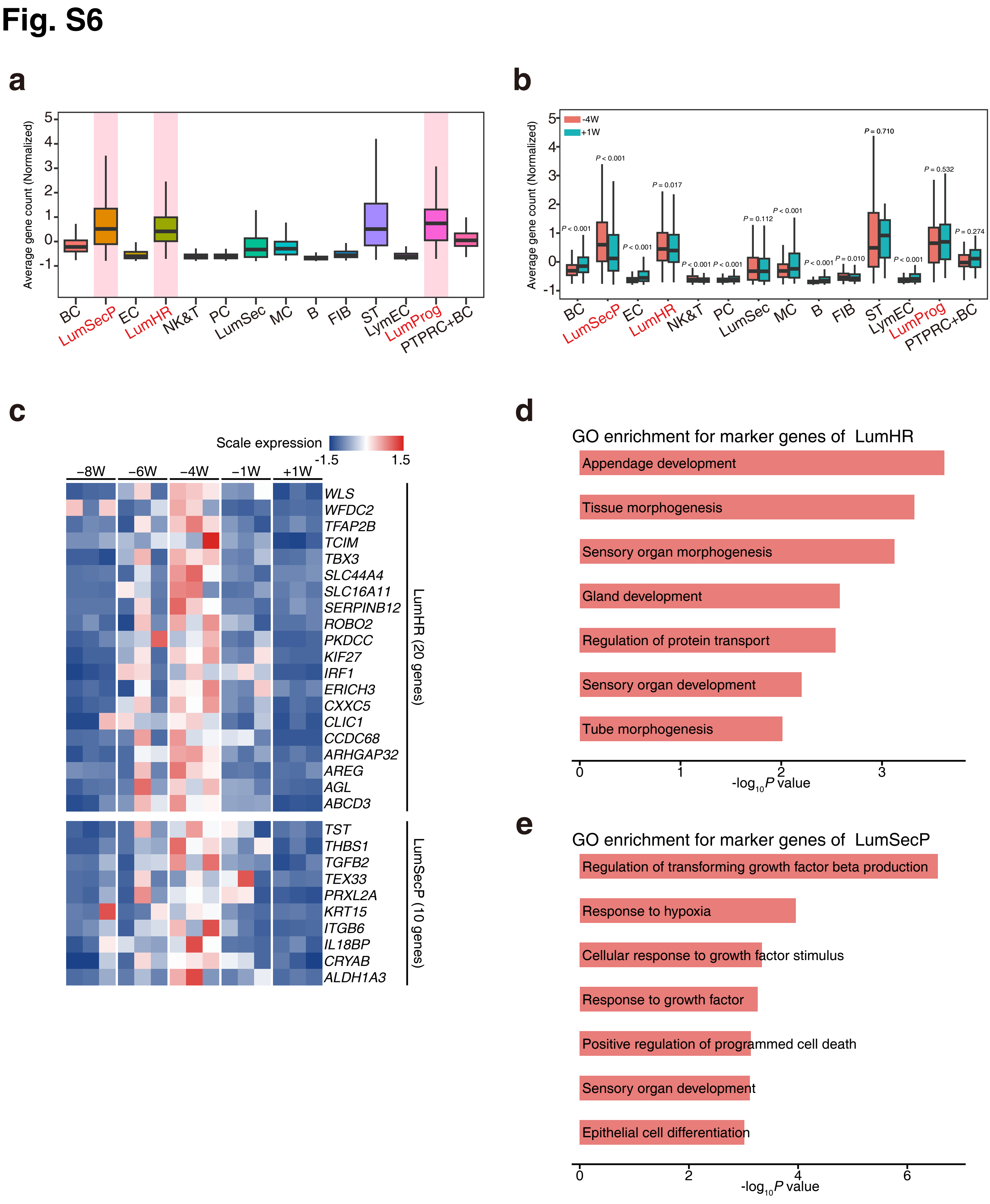
Fig. S6
a
Average gene count (Normalized)
BC
LumSecP
EC
LumHR
NK&T
PC
LumSec
MC
B
FIB
ST
LymEC
LumProg
PTPRC+BC
b
-4W
+1W
P < 0.001
P = 0.017
P = 0.112
P = 0.710
P = 0.532
P = 0.274
P = 0.010
c
Scale expression
-1.5
1.5
−8W
−6W
−4W
−1W
+1W
WLS
WFDC2
TFAP2B
TCIM
TBX3
SLC44A4
SLC16A11
SERPINB12
ROBO2
PKDCC
KIF27
IRF1
ERICH3
CXXC5
CLIC1
CCDC68
ARHGAP32
AREG
AGL
ABCD3
LumHR (20 genes)
TST
THBS1
TGFB2
TEX33
PRXL2A
KRT15
ITGB6
IL18BP
CRYAB
ALDH1A3
LumSecP (10 genes)
d
GO enrichment for marker genes of LumHR
Appendage development
Tissue morphogenesis
Sensory organ morphogenesis
Gland development
Regulation of protein transport
Sensory organ development
Tube morphogenesis
-log10P value
e
GO enrichment for marker genes of LumSecP
Regulation of transforming growth factor beta production
Response to hypoxia
Cellular response to growth factor stimulus
Response to growth factor
Positive regulation of programmed cell death
Sensory organ development
Epithelial cell differentiation
-log10P value

Fig. S7

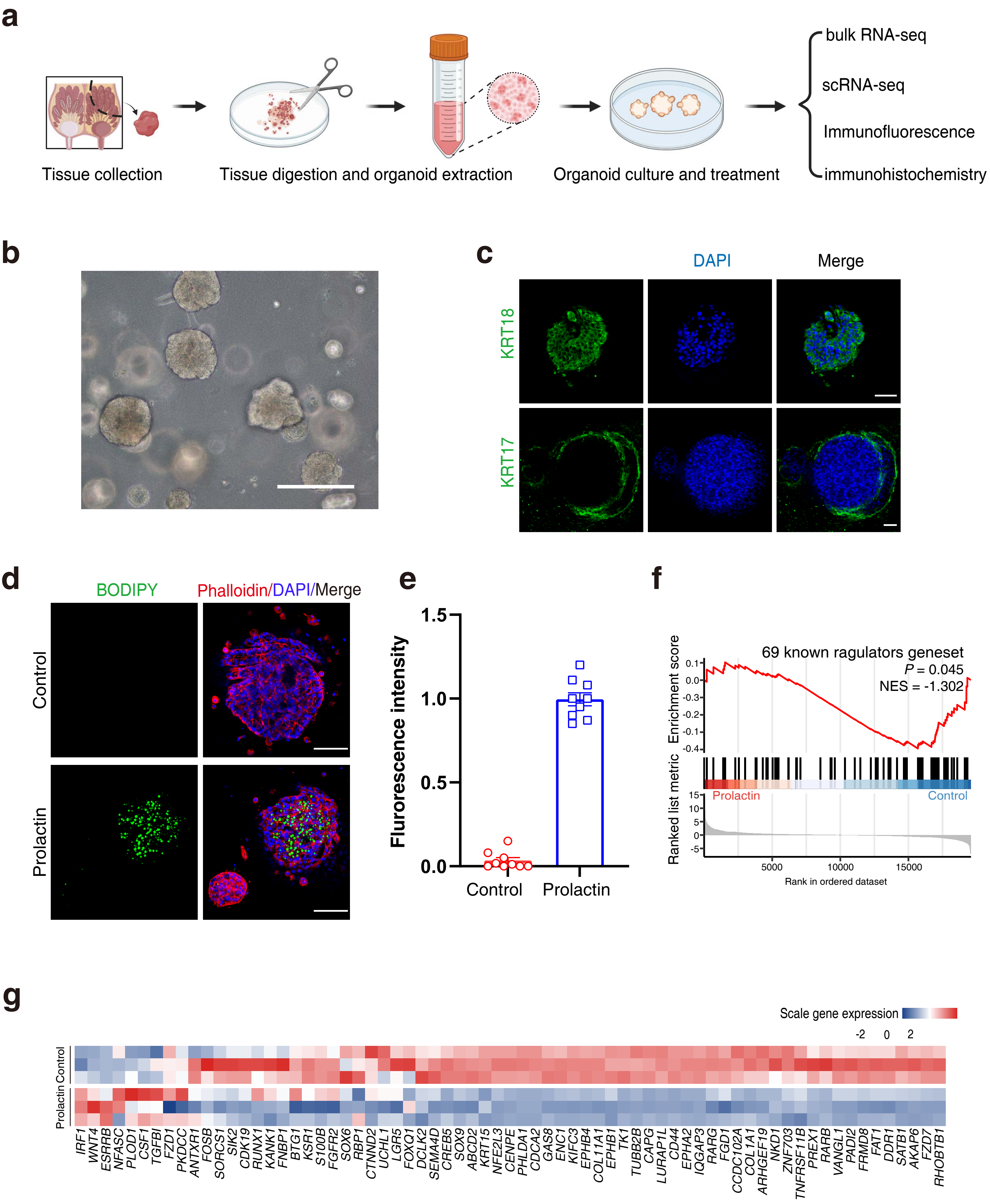

a
Tissue collection
Tissue digestion and organoid extraction
Organoid culture and treatment
bulk RNA-seq
scRNA-seq
Immunofluorescence
immunohistochemistry
b
c
DAPI
Merge
KRT18
KRT17
d
BODIPY
Phalloidin/DAPI/Merge
Control
Prolactin
e
Fluroescence intensity
1.5
1.0
0.5
0.0
Control
Prolactin
f
69 known ragulators geneset
P = 0.045
NES = -1.302
Enrichment score
Ranked list metric
Prolactin
Control
5000
10000
15000
Rank in ordered dataset
g
Scale gene expression
-2
0
2
Control
Prolactin
IRF1 WNT4 ESRRB NFASC PLOD1 CSF1 TGFBI FZD1 PKDCC ANTXR1 FOSB SORCS1 SIK2 CDK19 RUNX1 KANK1 FNBP1 BTG1 KSR1 S100B FGFR2 SOX6 RBP1 CTNND2 UCHL1 LGR5 FOXQ1 DCLK2 SEMA4D CREB5 SOX9 ABCD2 KRT15 NFE2L3 CENPE PHLDA1 CDCA2 GAS8 ENC1 KIFC3 EPHB4 COL11A1 EPHB1 TK1 TUBB2B CAPG LURAP1L CD44 EPHA2 IQGAP3 RARG FGD1 CCDC102A COL1A1 ARHGEF19 NKD1 ZNF703 TNFRSF11B PREX1 RARB VANGL1 PADI2 FRMD8 FAT1 DDR1 SATB1 AKAP6 FZD7 RHOBTB1

Fig.S8

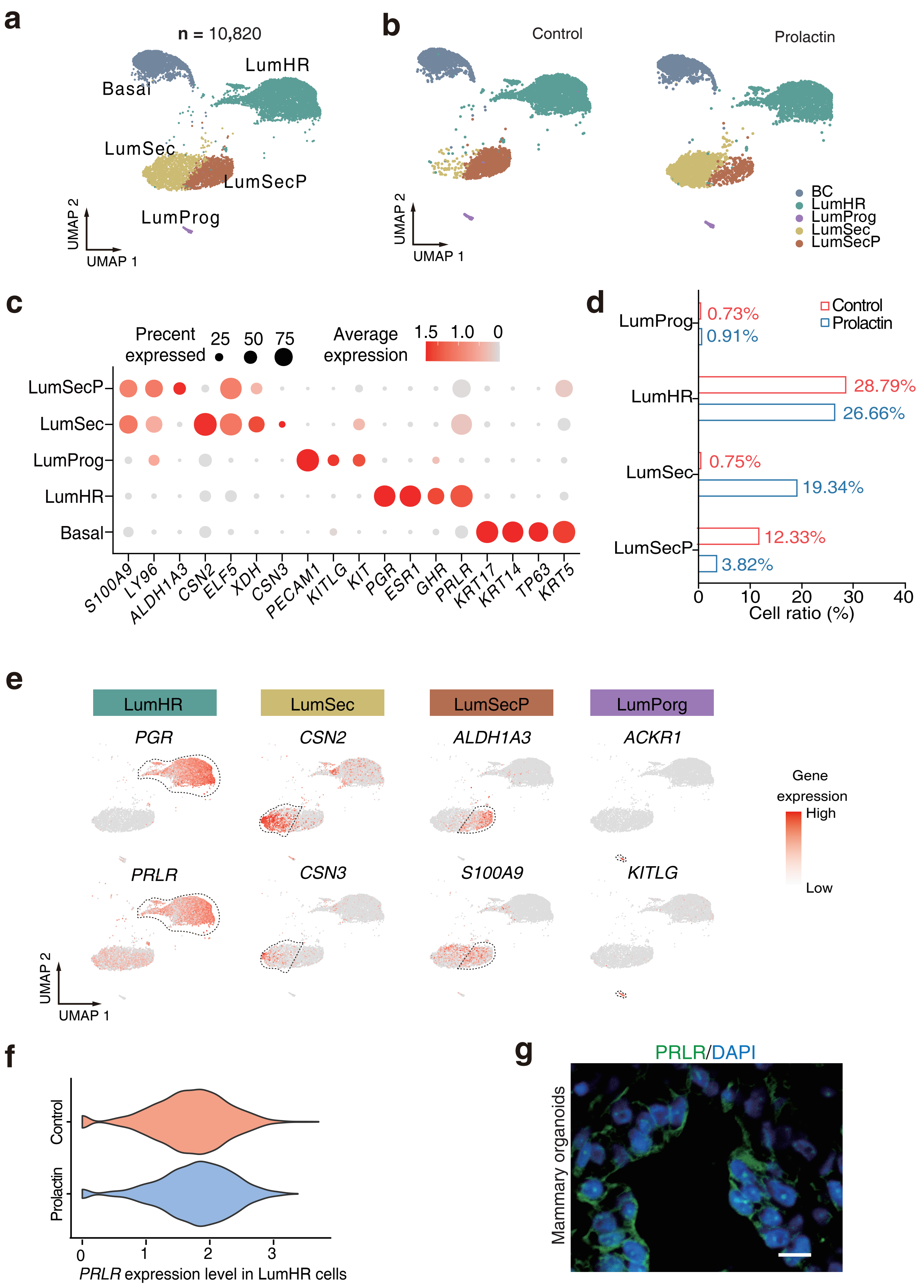
a
n = 10,820
Basal
LumHR
LumSec
LumSecP
LumProg
UMAP 2
UMAP 1
b
Control
Prolactin
BC
LumHR
LumProg
LumSec
LumSecP
c
Precent expressed
25 50 75
Average expression
1.5 1.0 0
LumSecP
LumSec
LumProg
LumHR
Basal
S100A9 LY96 ALDH1A3 CSN2 ELF5 XDH CSN3 PECAM1 KITLG KIT PGR ESR1 GHR PRLR KRT17 KRT14 TP63 KRT5
d
Control
Prolactin
0.73%
0.91%
28.79%
26.66%
0.75%
19.34%
12.33%
3.82%
Cell ratio (%)
e
LumHR
LumSec
LumSecP
LumPorg
PGR
CSN2
ALDH1A3
ACKR1
PRLR
CSN3
S100A9
KITLG
Gene expression
High
Low
f
Control
Prolactin
PRLR expression level in LumHR cells
g
PRLR/DAPI
Mammary organoids

Fig.S9

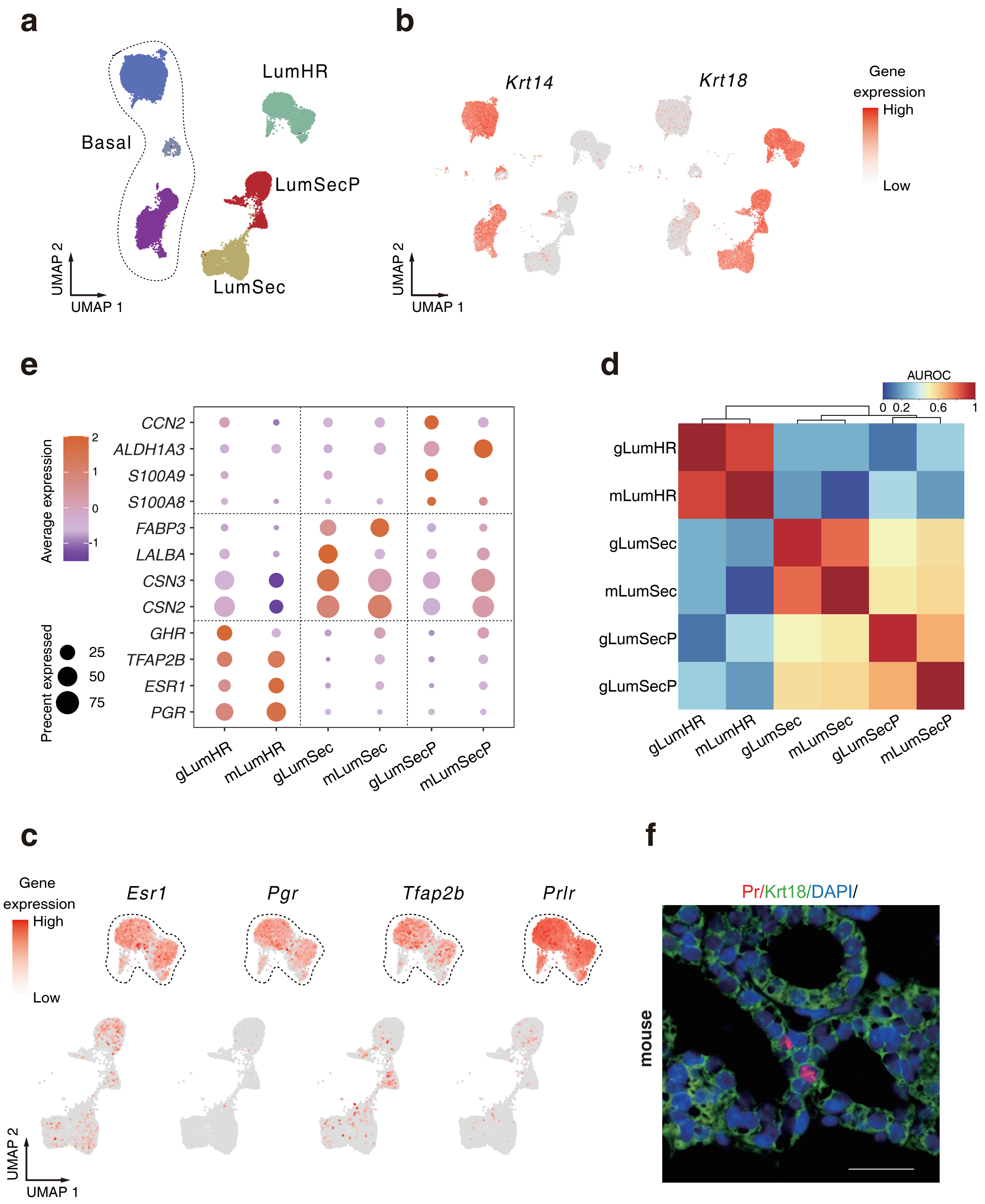

a
Basal
LumHR
LumSecP
LumSec
UMAP 2
UMAP 1
b
Krt14
Krt18
Gene expression
High
Low
UMAP 2
UMAP 1
e
Average expression
2
1
0
-1
Precent expressed
25
50
75
CCN2
ALDH1A3
S100A9
S100A8
FABP3
LALBA
CSN3
CSN2
GHR
TFAP2B
ESR1
PGR
gLumHR
mLumHR
gLumSec
mLumSec
gLumSecP
mLumSecP
d
AUROC
0
0.2
0.6
1
gLumHR
mLumHR
gLumSec
mLumSec
gLumSecP
gLumSecP
gLumHR
mLumHR
gLumSec
mLumSec
gLumSecP
mLumSecP
c
Gene expression
High
Low
Esr1
Pgr
Tfap2b
Prlr
UMAP 2
UMAP 1
f
Pr/Krt18/DAPI/
mouse

Fig.S10

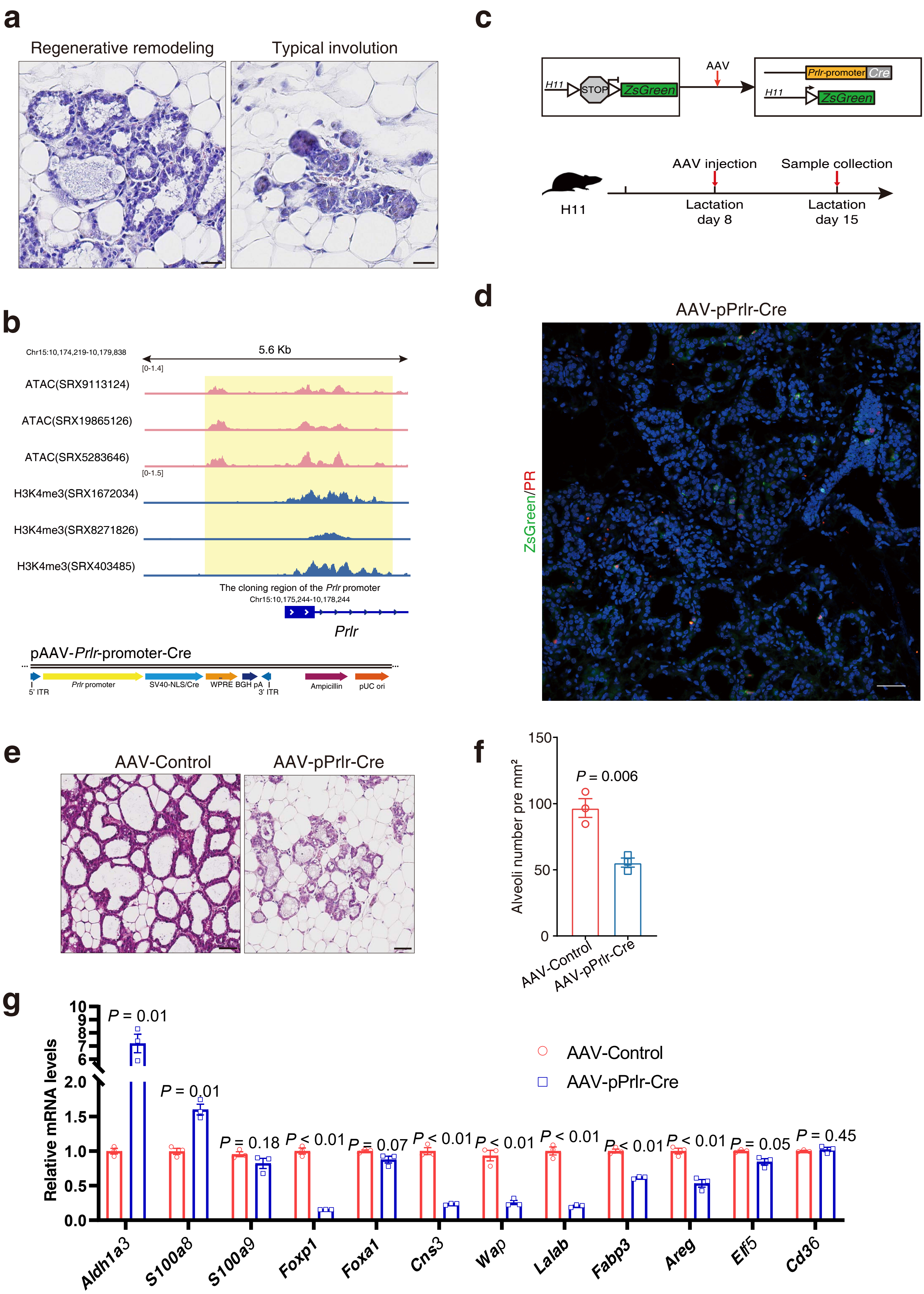

a
Regenerative remodeling
Typical involution
b
Chr15:10,174,219-10,179,838
5.6 Kb
[0-1.4]
ATAC(SRX9113124)
ATAC(SRX19865126)
ATAC(SRX5283646)
[0-1.5]
H3K4me3(SRX1672034)
H3K4me3(SRX8271826)
H3K4me3(SRX403485)
The cloning region of the Prlr promoter
Chr15:10,175,244-10,178,244
Prlr
pAAV-Prlr-promoter-Cre
5' ITR
Prlr promoter
SV40-NLS/Cre
WPRE BGH pA
3' ITR
Ampicillin
pUC ori
c
H11
STOP
ZsGreen
AAV
Prlr-promoter
Cre
H11
ZsGreen
AAV injection
Sample collection
H11
Lactation day 8
Lactation day 15
d
AAV-pPrlr-Cre
ZsGreen/PR
e
AAV-Control
AAV-pPrlr-Cre
f
P = 0.006
Alveoli number pre mm²
0
50
100
150
AAV-Control
AAV-pPrlr-Cre
g
Relative mRNA levels
P = 0.01
P = 0.01
P = 0.18
P < 0.01
P = 0.07
P < 0.01
P < 0.01
P < 0.01
P < 0.01
P < 0.01
P = 0.05
P = 0.45
AAV-Control
AAV-pPrlr-Cre
Aldh1a3
S100a8
S100a9
Foxp1
Foxa1
Cns3
Wap
Lalab
Fabp3
Areg
Elf5
Cd36

Fig. S11

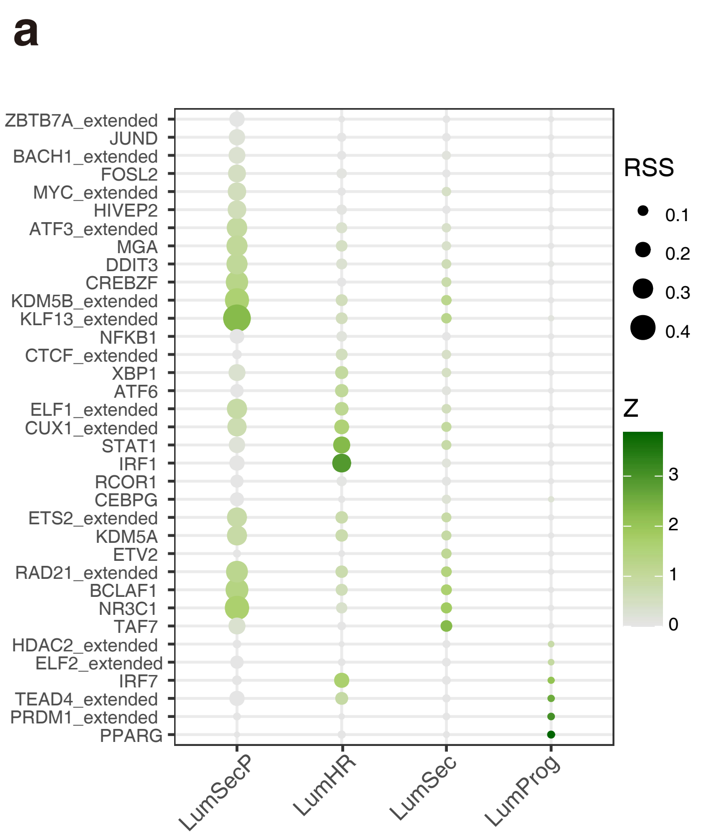
a
ZBTB7A_extended
JUND
BACH1_extended
FOSL2
MYC_extended
HIVEP2
ATF3_extended
MGA
DDIT3
CREBZF
KDM5B_extended
KLF13_extended
NFKB1
CTCF_extended
XBP1
ATF6
ELF1_extended
CUX1_extended
STAT1
IRF1
RCOR1
CEBPG
ETS2_extended
KDM5A
ETV2
RAD21_extended
BCLAF1
NR3C1
TAF7
HDAC2_extended
ELF2_extended
IRF7
TEAD4_extended
PRDM1_extended
PPARG
LumSecP
LumHR
LumSec
LumProg
RSS
0.1
0.2
0.3
0.4
Z
3
2
1
0

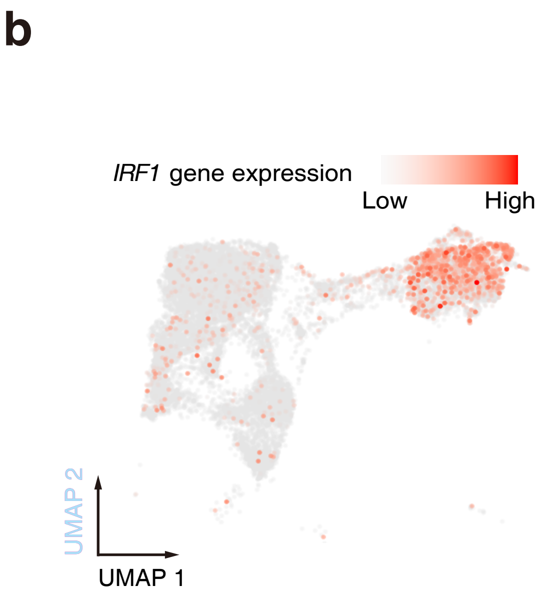
b
IRF1 gene expression
Low
High
UMAP 2
UMAP 1

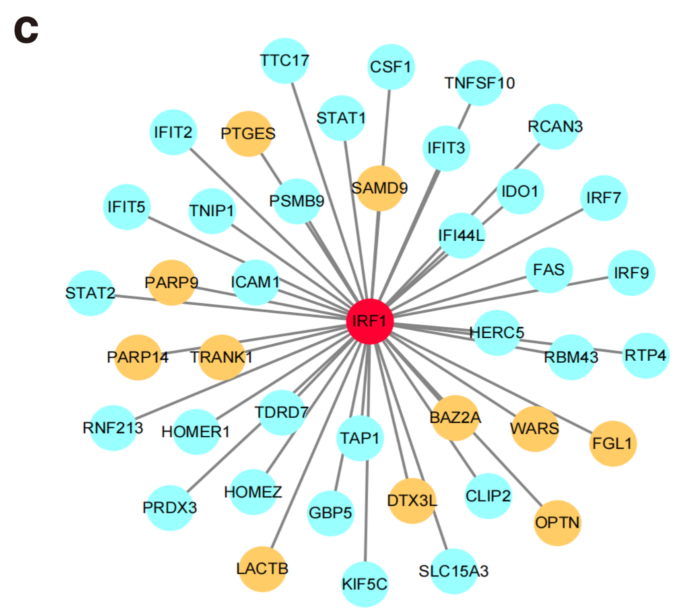
c
TTC17
CSF1
TNFSF10
IFIT2
PTGES
STAT1
RCAN3
IFIT3
SAMD9
IDO1
IFIT5
TNIP1
PSMB9
IRF7
IFI44L
FAS
IRF9
STAT2
PARP9
ICAM1
IRF1
HERC5
PARP14
TRANK1
RBM43
RTP4
TDRD7
BAZ2A
RNF213
HOMER1
TAP1
WARS
FGL1
HOMEZ
PRDX3
GBP5
DTX3L
CLIP2
OPTN
LACTB
KIF5C
SLC15A3

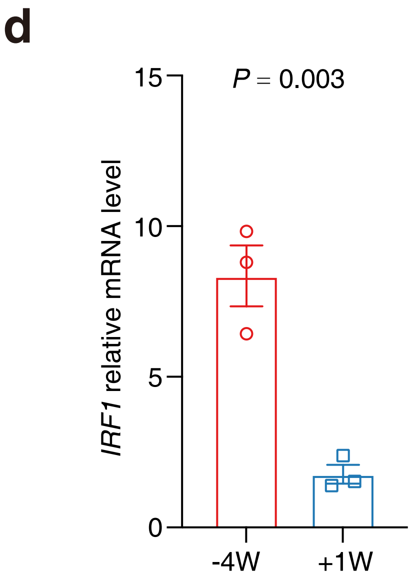
d
P = 0.003
IRF1 relative mRNA level
15
10
5
0
-4W
+1W

Fig. S12

a

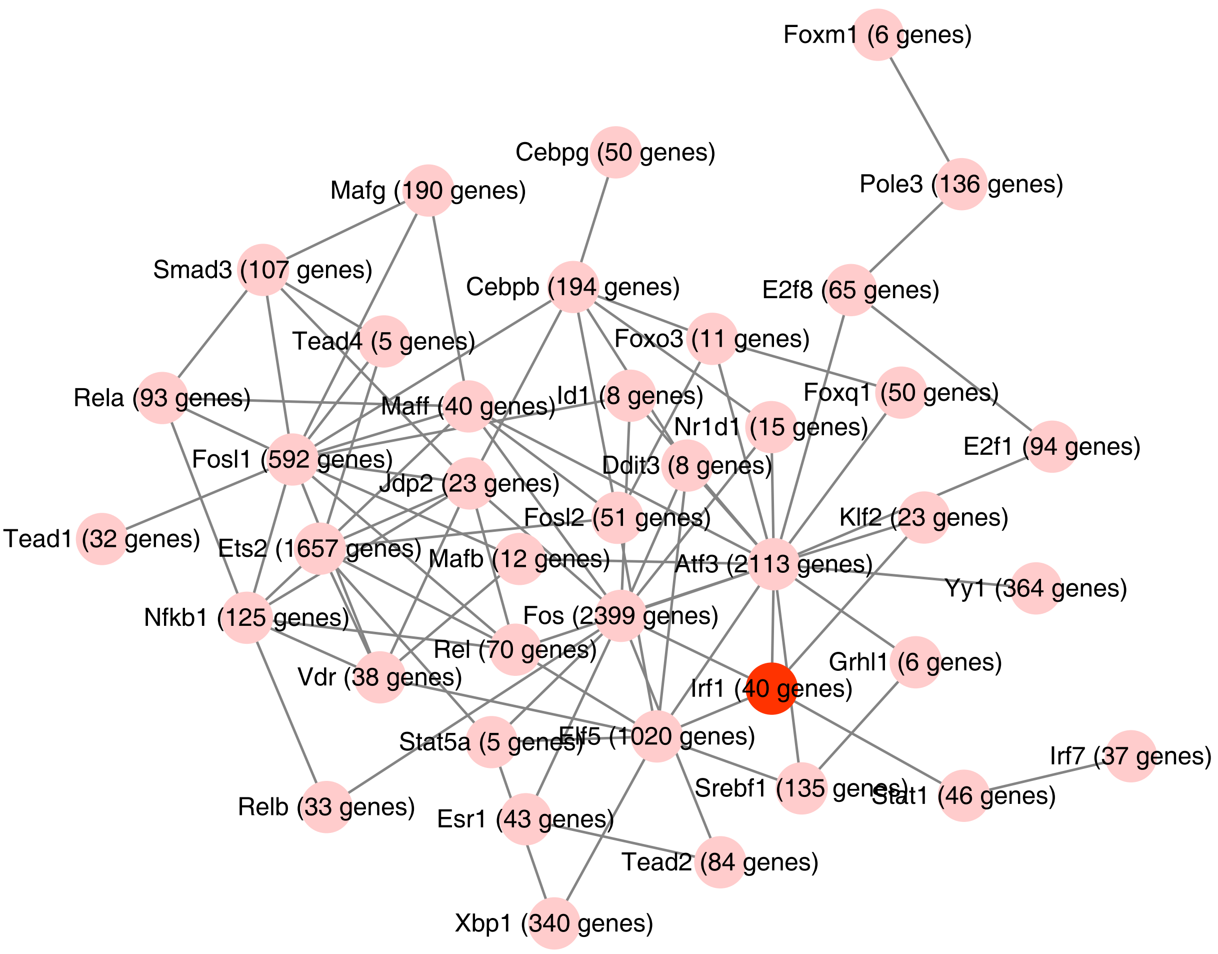
Foxm1 (6 genes)
Pole3 (136 genes)
Cebpg (50 genes)
Mafg (190 genes)
Smad3 (107 genes)
Cebpb (194 genes)
E2f8 (65 genes)
Tead4 (5 genes)
Foxo3 (11 genes)
Rela (93 genes)
Maff (40 genes)
Id1 (8 genes)
Foxq1 (50 genes)
Nr1d1 (15 genes)
E2f1 (94 genes)
Fosl1 (592 genes)
Ddit3 (8 genes)
Jdp2 (23 genes)
Fosl2 (51 genes)
Klf2 (23 genes)
Tead1 (32 genes)
Ets2 (1657 genes)
Mafb (12 genes)
Atf3 (2113 genes)
Yy1 (364 genes)
Nfkb1 (125 genes)
Fos (2399 genes)
Rel (70 genes)
Grhl1 (6 genes)
Vdr (38 genes)
Irf1 (40 genes)
Stat5a (5 genes)
Elf5 (1020 genes)
Irf7 (37 genes)
Srebf1 (135 genes)
Stat1 (46 genes)
Relb (33 genes)
Esr1 (43 genes)
Tead2 (84 genes)
Xbp1 (340 genes)

b

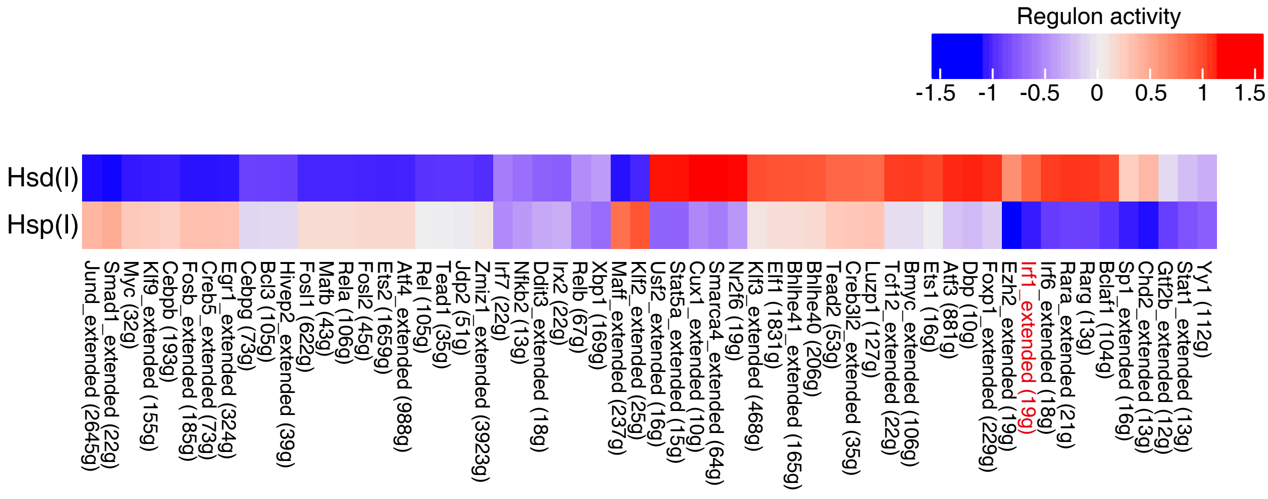
Regulon activity
-1.5
-1
-0.5
0
0.5
1
1.5
Hsd(I)
Hsp(I)
Jund_extended (2645g)
Smad1_extended (22g)
Myc (32g)
Klf9_extended (155g)
Cebpb (193g)
Fosb_extended (185g)
Creb5_extended (73g)
Egr1_extended (324g)
Cebpg (73g)
Bcl3 (105g)
Hivep2_extended (39g)
Fosl1 (622g)
Mafb (43g)
Rela (106g)
Fosl2 (45g)
Ets2 (1659g)
Atf4_extended (988g)
Rel (105g)
Tead1 (35g)
Jdp2 (51g)
Zmiz1_extended (3923g)
Irf7 (22g)
Nfkb2 (13g)
Ddit3_extended (18g)
Irx2 (22g)
Relb (67g)
Xbp1 (169g)
Maff_extended (237g)
Klf2_extended (25g)
Usf2_extended (16g)
Stat5a_extended (15g)
Cux1_extended (10g)
Smarca4_extended (64g)
Nr2f6 (19g)
Klf3_extended (468g)
Elf1 (1831g)
Bhlhe41_extended (165g)
Bhlhe40 (206g)
Tead2 (53g)
Creb3l2_extended (35g)
Luzp1 (127g)
Tcf12_extended (22g)
Bmyc_extended (106g)
Ets1 (16g)
Atf3 (881g)
Dbp (10g)
Foxp1_extended (229g)
Ezh2_extended (19g)
Irf1_extended (19g)
Irf6_extended (18g)
Rara_extended (21g)
Rarg (13g)
Bclaf1 (104g)
Sp1_extended (16g)
Chd2_extended (13g)
Gtf2b_extended (12g)
Stat1_extended (13g)
Yy1 (112g)

Fig. S13

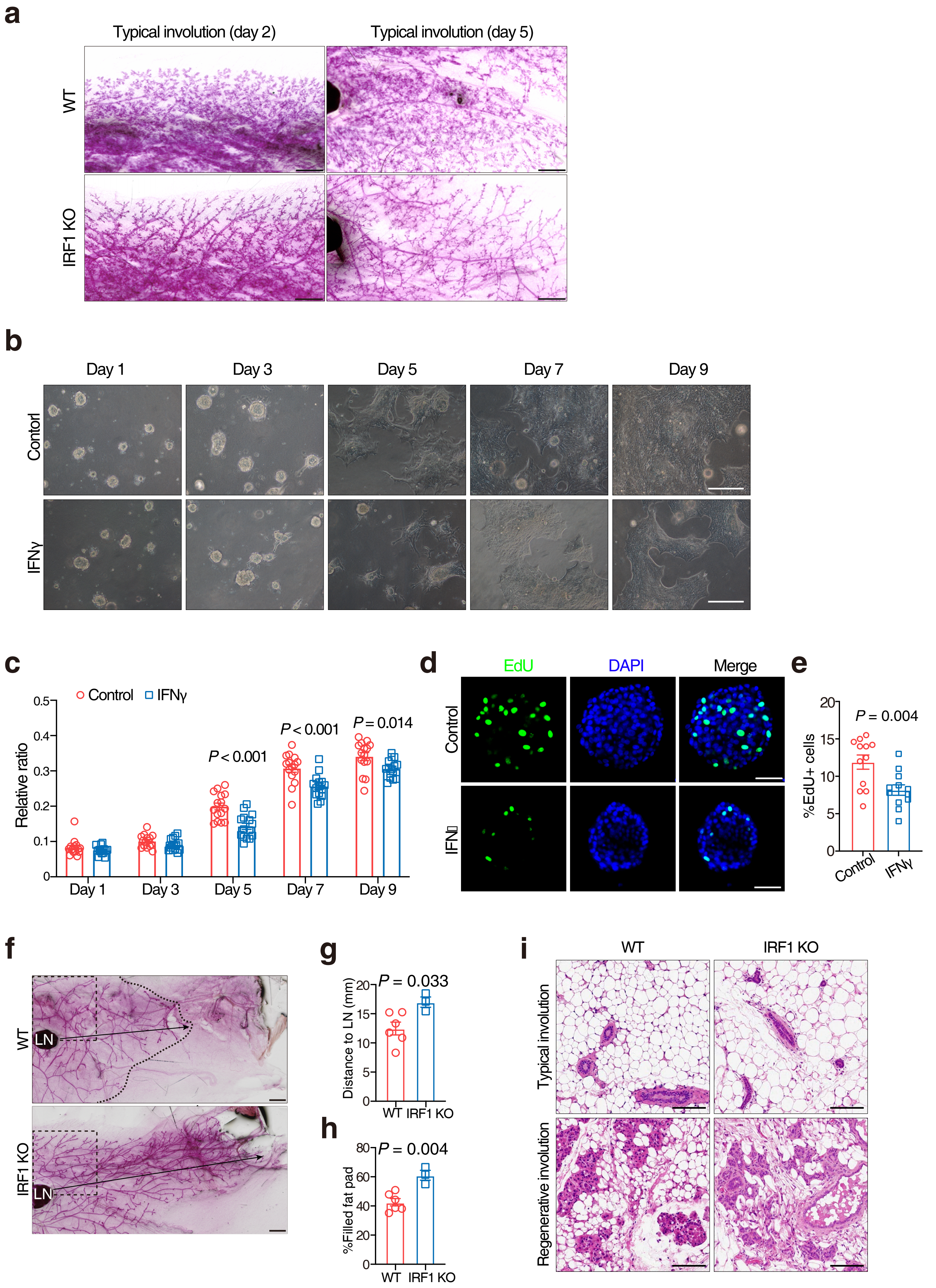

a
Typical involution (day 2)
Typical involution (day 5)
WT
IRF1 KO
b
Day 1
Day 3
Day 5
Day 7
Day 9
Contorl
IFNγ
c
Control
IFNγ
Relative ratio
P < 0.001
P < 0.001
P = 0.014
Day 1
Day 3
Day 5
Day 7
Day 9
d
EdU
DAPI
Merge
Control
e
%EdU+ cells
P = 0.004
Control
IFNγ
f
WT
IRF1 KO
LN
g
Distance to LN (mm)
P = 0.033
WT
IRF1 KO
h
%Filled fat pad
P = 0.004
WT
IRF1 KO
i
WT
IRF1 KO
Typical involution
Regenerative involution

Fig. S14

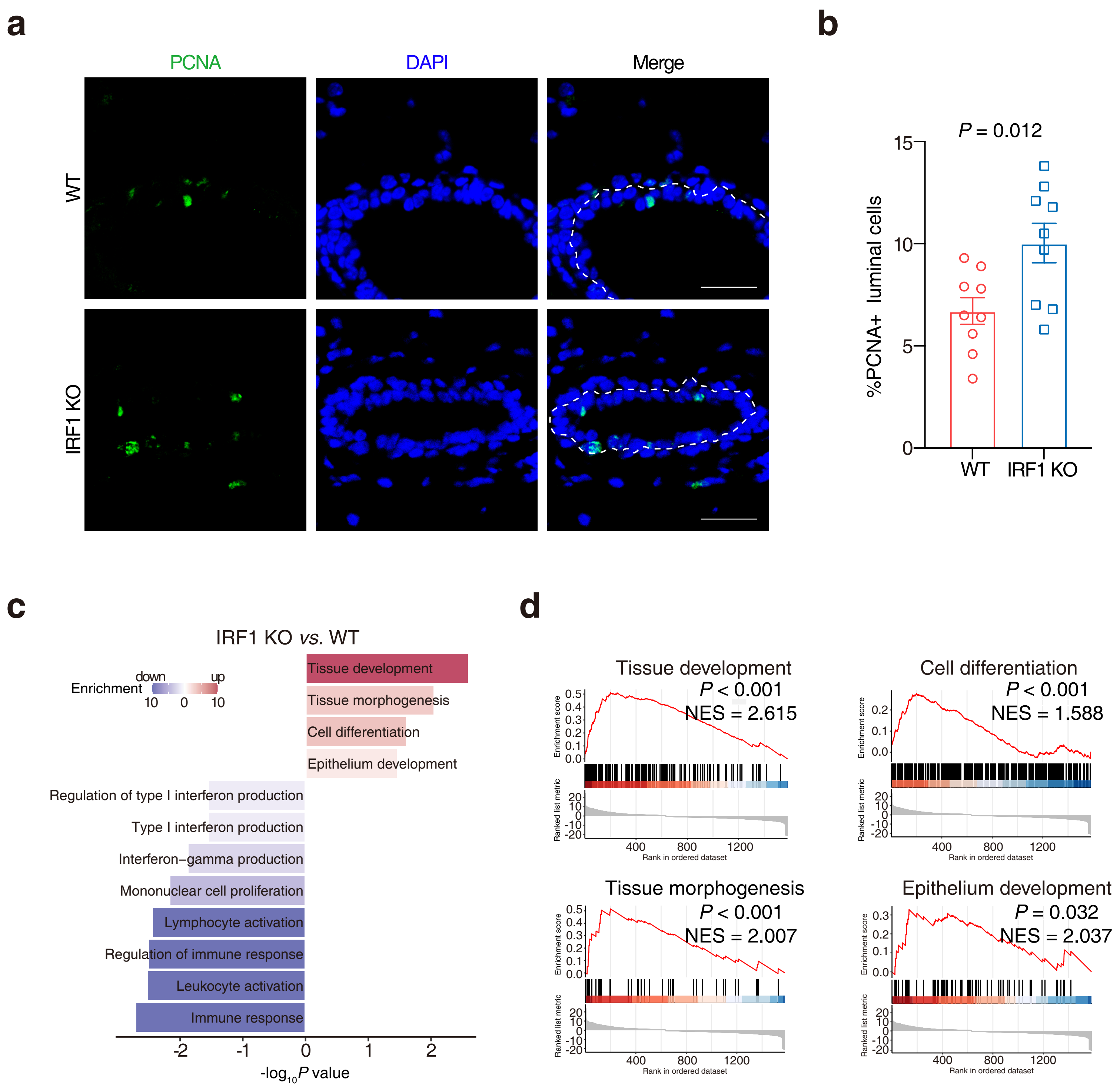

a
PCNA
DAPI
Merge
WT
IRF1 KO
b
P = 0.012
%PCNA+ luminal cells
15
10
5
0
WT
IRF1 KO
c
IRF1 KO vs. WT
Enrichment
down
up
10
0
10
Tissue development
Tissue morphogenesis
Cell differentiation
Epithelium development
Regulation of type I interferon production
Type I interferon production
Interferon−gamma production
Mononuclear cell proliferation
Lymphocyte activation
Regulation of immune response
Leukocyte activation
Immune response
-2
-1
0
1
2
-log10P value
d
Tissue development
P < 0.001
NES = 2.615
Cell differentiation
P < 0.001
NES = 1.588
Tissue morphogenesis
P < 0.001
NES = 2.007
Epithelium development
P = 0.032
NES = 2.037
Enrichment score
Ranked list metric
Rank in ordered dataset
400
800
1200

Fig. S15

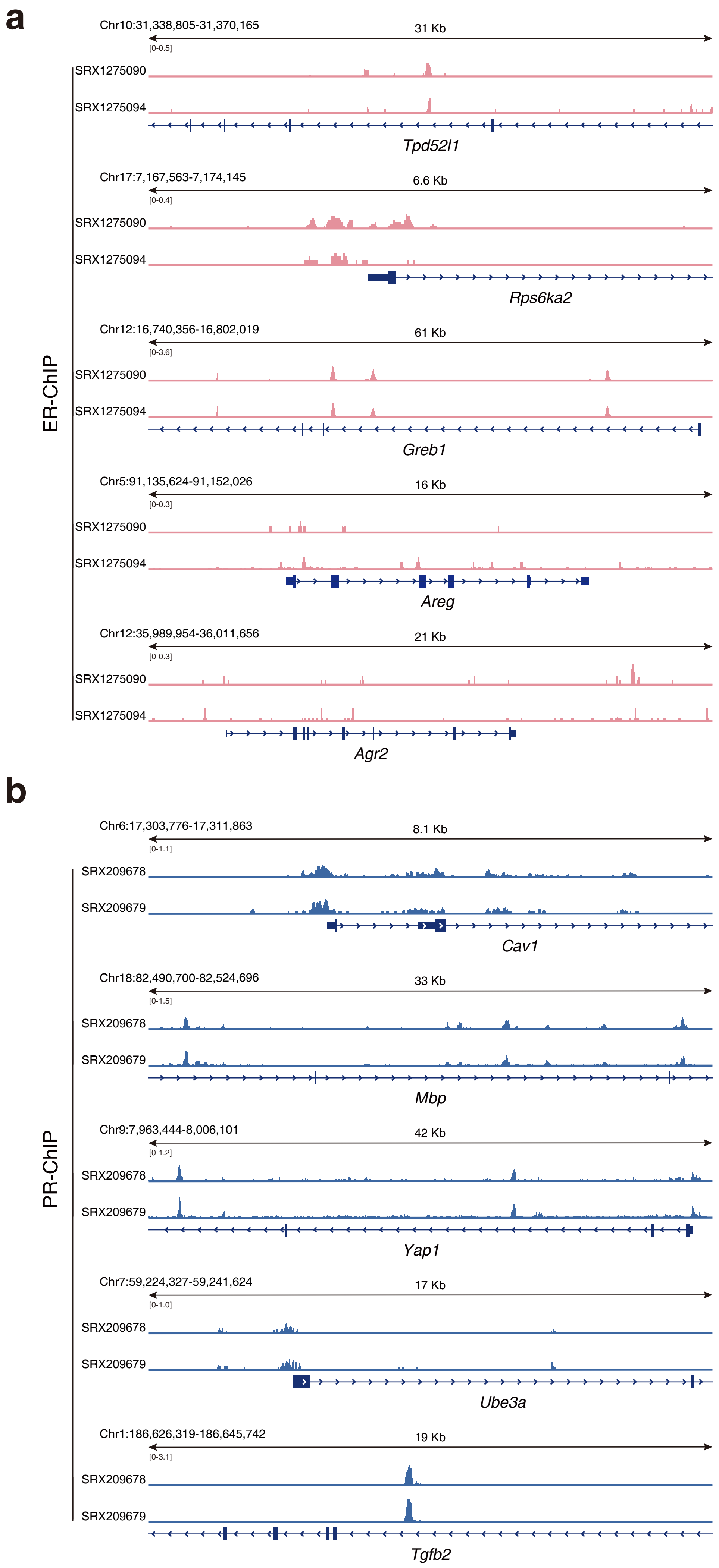
a
ER-ChIP
Chr10:31,338,805-31,370,165
31 Kb
[0-0.5]
SRX1275090
SRX1275094
Tpd52l1
Chr17:7,167,563-7,174,145
6.6 Kb
[0-0.4]
SRX1275090
SRX1275094
Rps6ka2
Chr12:16,740,356-16,802,019
61 Kb
[0-3.6]
SRX1275090
SRX1275094
Greb1
Chr5:91,135,624-91,152,026
16 Kb
[0-0.3]
SRX1275090
SRX1275094
Areg
Chr12:35,989,954-36,011,656
21 Kb
[0-0.3]
SRX1275090
SRX1275094
Agr2
b
PR-ChIP
Chr6:17,303,776-17,311,863
8.1 Kb
[0-1.1]
SRX209678
SRX209679
Cav1
Chr18:82,490,700-82,524,696
33 Kb
[0-1.5]
SRX209678
SRX209679
Mbp
Chr9:7,963,444-8,006,101
42 Kb
[0-1.2]
SRX209678
SRX209679
Yap1
Chr7:59,224,327-59,241,624
17 Kb
[0-1.0]
SRX209678
SRX209679
Ube3a
Chr1:186,626,319-186,645,742
19 Kb
[0-3.1]
SRX209678
SRX209679
Tgfb2

Fig. S16

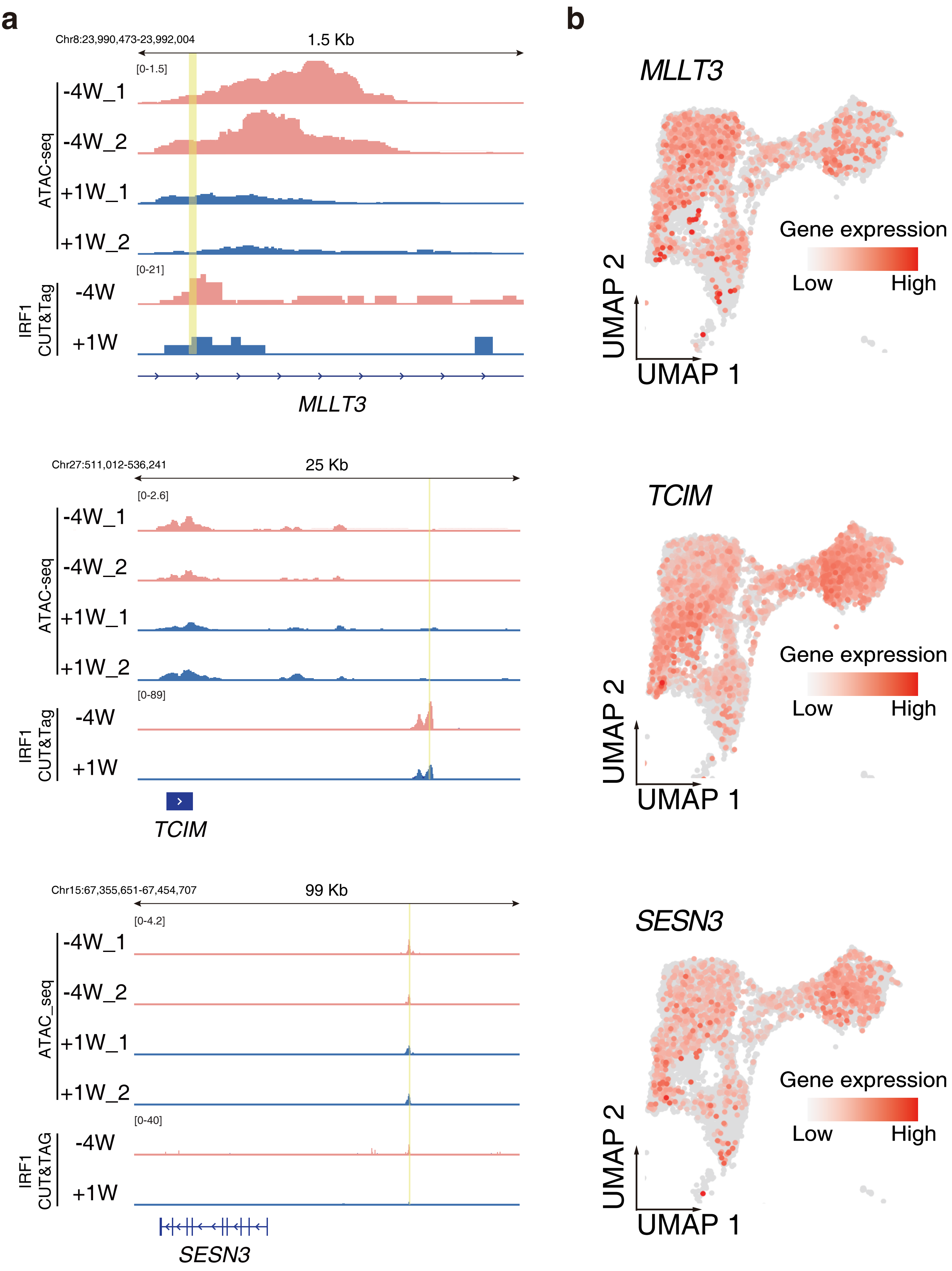

a
Chr8:23,990,473-23,992,004
1.5 Kb
[0-1.5]
ATAC-seq
-4W_1
-4W_2
+1W_1
+1W_2
[0-21]
IRF1 CUT&Tag
-4W
+1W
MLLT3
Chr27:511,012-536,241
25 Kb
[0-2.6]
ATAC-seq
-4W_1
-4W_2
+1W_1
+1W_2
[0-89]
IRF1 CUT&Tag
-4W
+1W
TCIM
Chr15:67,355,651-67,454,707
99 Kb
[0-4.2]
ATAC_seq
-4W_1
-4W_2
+1W_1
+1W_2
[0-40]
IRF1 CUT&TAG
-4W
+1W
SESN3
b
MLLT3
Gene expression
Low
High
UMAP 2
UMAP 1
TCIM
Gene expression
Low
High
UMAP 2
UMAP 1
SESN3
Gene expression
Low
High
UMAP 2
UMAP 1

Fig. S17

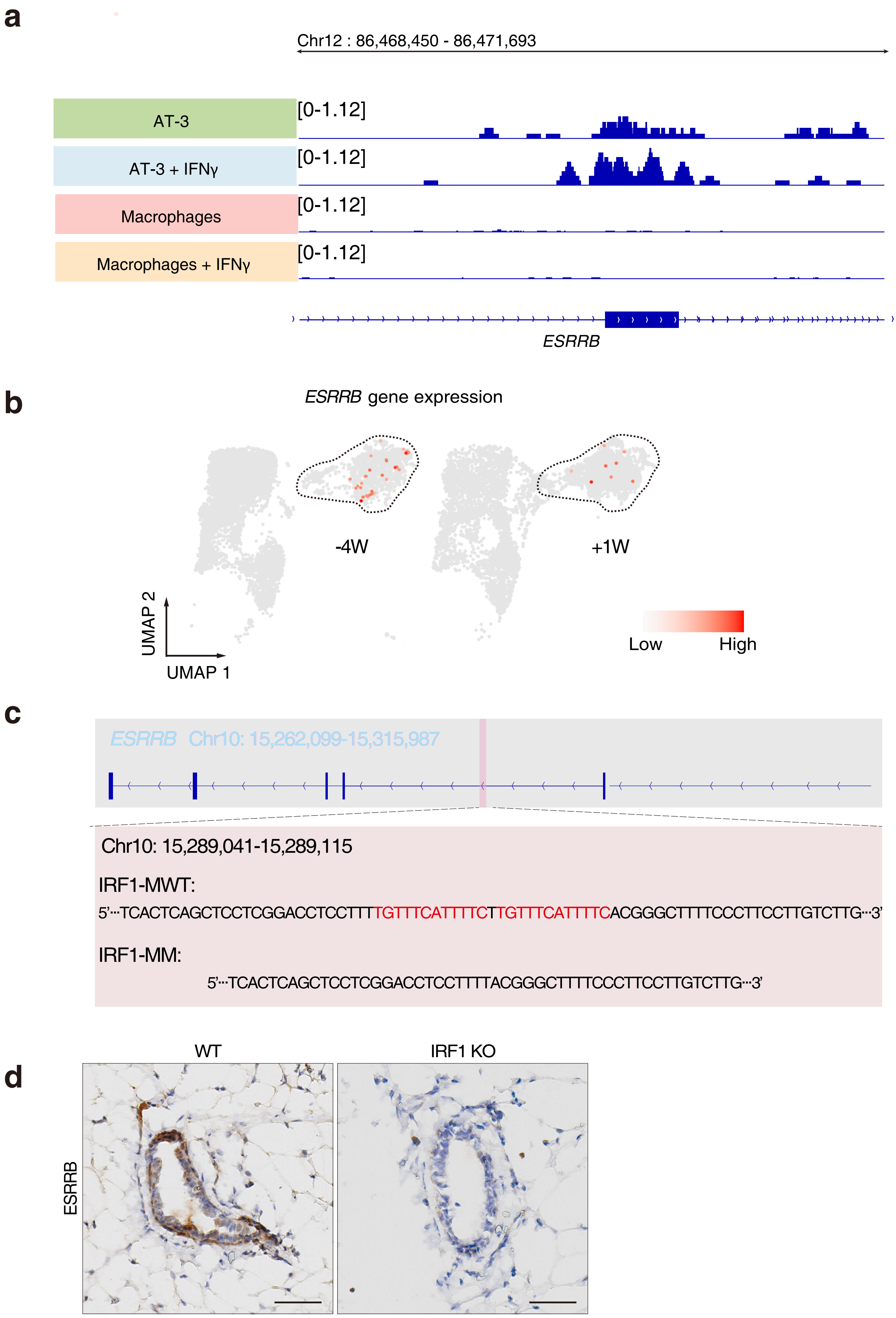
a
Chr12 : 86,468,450 - 86,471,693
AT-3
[0-1.12]
AT-3 + IFNγ
[0-1.12]
Macrophages
[0-1.12]
Macrophages + IFNγ
[0-1.12]
ESRRB
b
ESRRB gene expression
-4W
+1W
UMAP 2
UMAP 1
Low
High
c
ESRRB Chr10: 15,262,099-15,315,987
Chr10: 15,289,041-15,289,115
IRF1-MWT:
5'···TCACTCAGCTCCTCGGACCTCCTTTTGTTTCATTTTCTTGTTTCATTTTCACGGGCTTTTCCCTTCCTTGTCTTG···3'
IRF1-MM:
5'···TCACTCAGCTCCTCGGACCTCCTTTTACGGGCTTTTCCCTTCCTTGTCTTG···3'
d
WT
IRF1 KO
ESRRB

Legends for Supplementary Datasets S1 to S8

Dataset S1 (separate file). The gene expression level during RR at various time points (relative to +1W).

Dataset S2 (separate file). List of 187 RR associated genes and 69 known regulators related to cell proliferation and mammary gland morphogenesis.

Dataset S3 (separate file). GO analysis of 187 RR-associated genes. Similar terms with a high degree of redundancy were clustered.

Dataset S4 (separate file). Information of all barcodes and expression counts of cells in the scRNA-seq of goat mammary tissues.

Dataset S5 (separate file). List of gene markers for all cell types in goat mammary tissues.

Dataset S6 (separate file). Known gene markers used for cell identification.

Dataset S7 (separate file). Distribution of specific expression genes within different cell types.

Dataset S8 (separate file). List of all gene markers for basal and luminal cell types in goat mammary organoids.

Dataset S9 (separate file). Transcription factors are identified in the luminal subsets through SCENIC analysis.
